# Cytosolic dsDNA of mitochondrial origin induces cytotoxicity and neurodegeneration in cellular and zebrafish models of Parkinson’s disease

**DOI:** 10.1038/s41467-021-23452-x

**Published:** 2021-05-25

**Authors:** Hideaki Matsui, Junko Ito, Noriko Matsui, Tamayo Uechi, Osamu Onodera, Akiyoshi Kakita

**Affiliations:** 1 Department of Neuroscience of Disease, Brain Research Institute, Niigata University, Niigata, Japan; 2 Department of Neuroscience of Disease, Center for Transdisciplinary Research, Niigata University, Niigata, Japan; 3 Department of Pathology, Brain Research Institute, Niigata University, Niigata, Japan; 4 Frontier Science Research Center, University of Miyazaki, Miyazaki, Japan; 5 Department of Neurology, Brain Research Institute, Niigata University, Niigata, Japan

**Keywords:** Mitochondria, Parkinson's disease

## Abstract

Mitochondrial dysfunction and lysosomal dysfunction have been implicated in Parkinson’s disease (PD), but the links between these dysfunctions in PD pathogenesis are still largely unknown. Here we report that cytosolic dsDNA of mitochondrial origin escaping from lysosomal degradation was shown to induce cytotoxicity in cultured cells and PD phenotypes in vivo. The depletion of PINK1, GBA and/or ATP13A2 causes increases in cytosolic dsDNA of mitochondrial origin and induces type I interferon (IFN) responses and cell death in cultured cell lines. These phenotypes are rescued by the overexpression of DNase II, a lysosomal DNase that degrades discarded mitochondrial DNA, or the depletion of IFI16, which acts as a sensor for cytosolic dsDNA of mitochondrial origin. Reducing the abundance of cytosolic dsDNA by overexpressing human DNase II ameliorates movement disorders and dopaminergic cell loss in gba mutant PD model zebrafish. Furthermore, IFI16 and cytosolic dsDNA puncta of mitochondrial origin accumulate in the brain of patients with PD. These results support a common causative role for the cytosolic leakage of mitochondrial DNA in PD pathogenesis.

## Abstract (web summary)

Mitochondrial and lysosomal dysfunction are central to Parkinson’s disease (PD) pathogenesis. Here, the authors show mitochondrial dsDNA in the cytosol in cellular and Zebrafish models of PD induces cytotoxicity and neurodegeneration; knock-down of IFI16, a cytosolic dsDNA sensor, rescues cytotoxicity, as does overexpression of lysosomal DNAse II.

## Introduction

Mitochondrial dysfunction has been implicated in Parkinson’s disease (PD), and mutations in genes encoding mitochondrial proteins, including Parkin and PTEN-induced kinase 1 (PINK1), cause familial PD^1,2^. Autophagy and lysosomal dysfunction are also common characteristics of PD models and patients^3–5^. However, the links between mitochondrial dysfunction and autophagy–lysosome dysfunction in PD pathogenesis are still largely unknown. Damaged mitochondria are degraded by the autophagy–lysosome system, but insufficient degradation of mitochondrial components occurs in response to massive damage to the mitochondria and/or autophagy–lysosome dysfunction. In particular, mitochondrial DNA contains inflammatogenic unmethylated CpG motifs, and cytosolic dsDNA of mitochondrial origin is highly toxic^6,7^. In this work, cytosolic dsDNA of mitochondrial origin escaping from lysosomal degradation was shown to induce cytotoxicity and neurodegeneration in cellular and zebrafish models of PD.

## Results

### Depletion of some PARK gene products induces cytosolic dsDNA deposits of mitochondrial origin in cultured cells

**Fig. 1:**
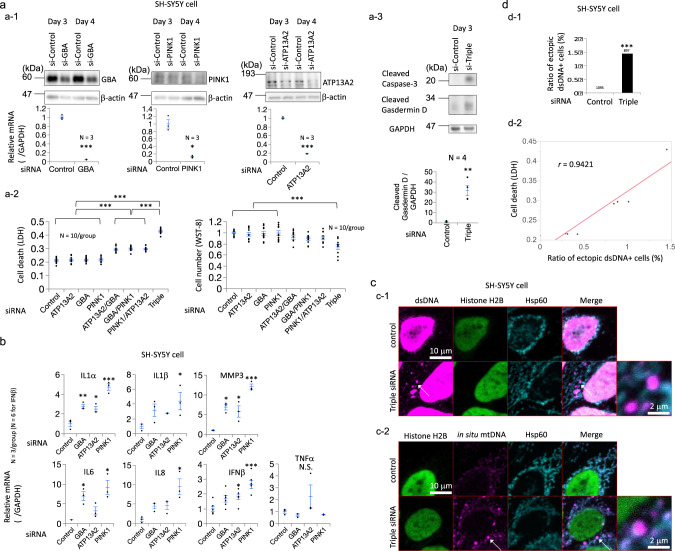
Loss of GBA, ATP13A2, and/or PINK1 leads to cytosolic leakage of mitochondrial DNA and cell death. **a** (**a-1**) Knockdown of GBA, ATP13A2, and/or PINK1 expression with siRNAs in SH-SY5Y cells. siRNA-mediated knockdown was confirmed at the mRNA and protein (western blotting) levels. **a-2** The death of GBA-, ATP13A2- and/or PINK1-depleted cells was determined using the LDH assay and WST-8 assay. Triple: Knockdown of GBA, ATP13A2, and PINK1 expression with siRNAs. **a-3** The death of GBA-, ATP13A2- and/or PINK1-depleted cells was determined by western blotting targeting cleaved Caspase-3 and cleaved Gasdermin D. Triple: Knockdown of GBA, ATP13A2 and PINK1 expression with siRNAs. **b** qPCR analysis of *IL-1α, IL-1β, MMP-3, IL-6, IL-8, IFN-β* and *TNF-α* mRNAs in GBA-, ATP13A2- or PINK1-depleted SH-SY5Y cells. N.S.: statistically not significant. **c** (**c-1**) Immunostaining for dsDNA, histone H2B and Hsp60 in SH-SY5Y cells transfected with GBA, ATP13A2, and PINK1 siRNAs. White arrows indicate cytosolic dsDNA of mitochondrial origin. Triple siRNA: Knockdown of GBA, ATP13A2, and PINK1 expression with siRNAs. **c-2** In situ hybridization of mitochondrial DNA and coimmunostaining for histone H2B and Hsp60 in SH-SY5Y cells transfected with GBA, ATP13A2, and PINK1 siRNAs. White arrows indicate cytosolic dsDNA of mitochondrial origin. Triple siRNA: Knockdown of GBA, ATP13A2, and PINK1 expression with siRNAs. **d** (**d-1**) The bar graph shows the ratio of ectopic dsDNA+ cells among SH-SY5Y cells transfected with GBA, ATP13A2, and PINK1 siRNAs. Triple: Knockdown of GBA, ATP13A2, and PINK1 expression with siRNAs. **d-2** The scatter plot shows the correlation between the ratio of ectopic dsDNA+ cells and cell death. The linear regression curve is shown as a red line. Cell death (LDH) values were derived from **a-2** and the ectopic dsDNA + cell ratio was assessed in the same way as **d-1**. The statistical details are described in Supplementary Table 4. Source data of Fig. 1 are provided as a Source Data file.

First, we examined the presence of cytosolic dsDNA of mitochondrial origin in cultured cells. In SH-SY5Y neuroblastoma cells, the depletion of PINK1, ATP13A2, and/or GBA resulted in cell death, as demonstrated by elevated LDH levels, decreased cell viability, and increased levels of the cleaved form of caspase-3 or Gasdermin D (Fig. 1a and Supplementary Fig. 1a, b). Type I IFN responses were simultaneously activated, including increases in the levels of *IL-1α, IL-1β, IL-6, IL-8, MMP-3,* and *IFN-β* mRNAs (Fig. 1b). Cytosolic dsDNA deposits were detected using an anti-dsDNA antibody, and the number of cytosolic dsDNA deposits was positively correlated with cell death (Fig. 1c, d). The increased cytosolic dsDNA content did not colocalize with histone H2B and included mitochondrial DNA sequences, as demonstrated by in situ hybridization (Fig. 1c), indicating that the cytosolic dsDNA was derived from the mitochondria. These dsDNA puncta rarely colocalized with the lysosome marker cathepsin D or with autophagosomes, and some of the dsDNA appeared to leak out of the mitochondria (Supplementary Fig. 1c). Using HeLa cells and different kinds of siRNAs, we reproduced the phenotypes induced by the depletion of GBA or ATP13A2, including increased levels of the cleaved form of Gasdermin D (Supplementary Fig. 1d, e), decreased cell viability (Supplementary Fig. 1f), elevated type I IFN responses (Supplementary Fig. 1g) and cytosolic dsDNA deposits of mitochondrial origin (Supplementary Fig. 1h). The increases in cytosolic dsDNA of mitochondrial origin in GBA- and ATP13A2-depleted HeLa cells were further supported by qPCR analysis of the cytosolic fraction of these cells (Supplementary Fig. 1i).

There seem to be two possibilities whereby cytosolic dsDNA of mitochondrial origin may induce inflammatory responses: cytosolic dsDNA of mitochondrial origin may activate type I IFN responses within the original cell (cell-autonomous action), or cytosolic dsDNA of mitochondrial origin may leak outside of the original cell and then stimulate cell surface receptors (non-cell-autonomous action). To discriminate between these possibilities, mitochondrial DNA was intracellularly introduced to SH-SY5Y cells or was extracellularly added to the cell medium. One day after the intracellular introduction of mitochondrial DNA, type I IFN responses were activated, including increases in the levels of *IL-1α, IL-1β, IL-6, IL-8,* and *IFN-β* mRNAs (Supplementary Fig. 1j). Although the same amount of mitochondrial DNA was used, the activation of type I IFN responses was not observed when mitochondrial DNA was added to the cell culture medium (Supplementary Fig. 1j). These results suggest that cytosolic dsDNA of mitochondrial origin exerts type I IFN responses within the original cell in a cell-autonomous manner.

### DNase II can rescue the phenotypes induced by cytosolic dsDNA of mitochondrial origin in cultured cells

**Fig. 2:**
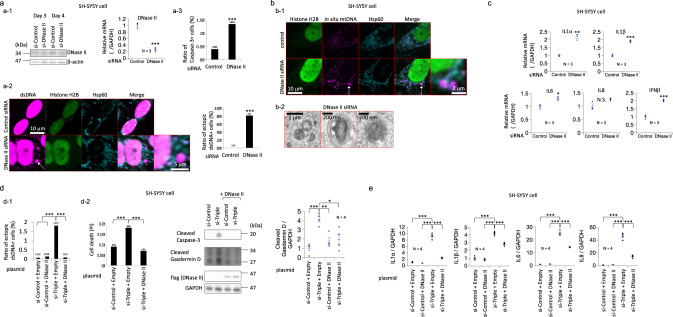
Effects of DNase II on the cytosolic accumulation of mitochondrial DNA and cell death. **a** (**a-1**) DNase II knockdown (6 days after transfection) in SH-SY5Y cells. siRNA-mediated knockdown was confirmed at the mRNA and protein (western blotting) levels. **a-2** Immunostaining for dsDNA, histone H2B, and Hsp60 in SH-SY5Y cells transfected with the DNase II siRNA shows cytosolic dsDNA deposits (white arrow). The bar graph shows the ratio of ectopic dsDNA+ cells among SH-SY5Y cells transfected with DNase II siRNAs. **a-3** The death of DNase II-depleted cells was assayed by immunofluorescence staining for caspase-3. **b** (**b-1**) In situ hybridization of mitochondrial DNA and coimmunostaining for histone H2B and Hsp60 in SH-SY5Y cells transfected with DNase II siRNA. A white arrow indicates cytosolic dsDNA of mitochondrial origin. **b-2** Transmission electron micrographs of SH-SY5Y cells transfected with the DNase II siRNA. **c** qPCR analysis of *DNase II, IL-1α, IL-1β, IL-6, IL-8,* and *IFN-β* mRNAs in DNase II-depleted SH-SY5Y cells. N.S.: statistically not significant. **d** Effects of DNase II overexpression on the death of SH-SY5Y cells with GBA, ATP13A2, and PINK1 knockdown. **d-1** The increase in cytosolic dsDNA deposits in GBA-, ATP13A2-, and PINK1-depleted SH-SY5Y cells were suppressed by DNase II overexpression. si-Triple: Knockdown of GBA, ATP13A2, and PINK1 expression with siRNAs. **d-2** Cell death and rescue effects were assayed using western blotting for cleaved Caspase-3 and cleaved Gasdermin D and propidium iodide (PI) staining. si-Triple: Knockdown of GBA, ATP13A2, and PINK1 expression with siRNAs. **e** Effects of DNase II overexpression on the type I IFN responses of SH-SY5Y cells with GBA, ATP13A2, and PINK1 knockdown. si-Triple: Knockdown of GBA, ATP13A2, and PINK1 expression with siRNAs. The statistical details are described in Supplementary Table 4. Source data of Fig. 2 are provided as a Source Data file.

DNase II is an acid DNase expressed in the lysosome, and DNase II digests mitochondrial DNA in the autophagy–lysosome system^8–10^. The depletion of DNase II in SH-SY5Y cells resulted in cell death, elevated levels of the mRNAs of genes involved in type I IFN responses, and a marked increase in the number of cytosolic dsDNA deposits (Fig. 2a–c and Supplementary Fig. 2a, b). The depletion of DNase II in HeLa cells resulted in similar phenotypes, including cell death, type I IFN responses, and increased deposition of cytosolic dsDNA of mitochondrial origin (Supplementary Fig. 2c–e). According to the electron microscopy data, the decrease in DNase II expression induced the formation of lysosome-like bodies that contained granular deposits and membranous structures (Fig. 2b). The depletion of PINK1, ATP13A2, and GBA resulted in the death of SH-SY5Y cells, while the concurrent overexpression of DNase II rescued cell death (Fig. 2d and e and Supplementary Fig. 2f). As expected, DNase II overexpression significantly reduced the number of cytosolic dsDNA deposits and type I IFN responses in PINK1-, ATP13A2- and GBA-depleted SH-SY5Y cells (Fig. 2d, e). Cell death and type I IFN responses in GBA- or ATP13A2-depleted HeLa cells were also reduced by DNase II overexpression (Supplementary Fig. 2g–i). Taken together, the results indicated that DNase II overexpression increased the degradation of dsDNA of mitochondrial origin and reduced the cell death and type I IFN responses caused by the cytosolic dsDNA of mitochondrial origin.

### IFI16 acts as a sensor of cytosolic dsDNA of mitochondrial origin

**Fig. 3:**
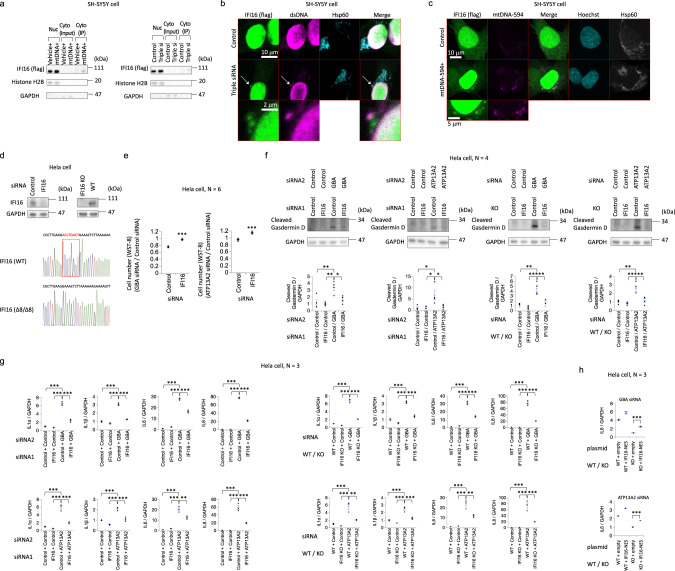
Effects of IFI16 on the cytotoxicity induced by cytosolic dsDNA of mitochondrial origin. **a** SH-SY5Y cells in which mitochondrial DNA was introduced (left figures) or GBA, ATP13A2, and PINK1 were depleted (right figures) were subjected to immunoprecipitation using an anti-dsDNA antibody. The immunoprecipitation results show an interaction between cytosolic dsDNA of mitochondrial origin and flag-tagged IFI16 (IFI16-flag). Nuc: Nuclear fraction. Cyto: Cytosolic fraction. Triple si: Knockdown of GBA, ATP13A2, and PINK1 expression with siRNAs. IP: Immunoprecipitation. **b** Colocalization of cytosolic dsDNA and IFI-16-flag in GBA-, ATP13A2-, and PINK1-depleted SH-SY5Y cells. Triple siRNA: Knockdown of GBA, ATP13A2, and PINK1 expression with siRNAs. **c** Colocalization of exogenous mitochondrial DNA conjugated with Alexa Fluor 594 and IFI-16-flag in SH-SY5Y cells. mtDNA-594: mitochondrial DNA conjugated with Alexa Fluor 594. **d** siRNA knockdown of IFI16 in HeLa cells and establishment of IFI16 knockout HeLa cells. Western blotting targeting endogenous IFI16 and the genome sequence show a homologous 8 bp deletion resulting in the complete loss of the IFI16 protein. WT: Wild type. **e** Effect of IFI16 depletion on cell viability (WST-8 assay) in GBA- or ATP13A2-depleted HeLa cells. **f** Effect of IFI16 depletion (siRNA knockdown or knockout) on cleaved Gasdermin D in HeLa cells with GBA or ATP13A2 knockdown. **g** Effect of IFI16 depletion (siRNA knockdown or knockout) on type I IFN responses in HeLa cells with GBA or ATP13A2 knockdown. qPCR results for *IL-1α, IL-1β, IL-6,* and *IL-8* mRNAs are shown. **h** Effect of IFI16 overexpression in the cytosol (MAPKK nuclear export signal (NES)-tagged IFI16) on type I IFN responses in IFI16 knockout HeLa cells with GBA or ATP13A2 knockdown. qPCR results for *IL-6* mRNAs are shown. The statistical details are described in Supplementary Table 4. Source data of Fig. 3 are provided as a Source Data file.

Next, we attempted to identify the sensor protein for cytosolic dsDNA of mitochondrial origin. Mitochondria are believed to be derived from the circular genomes of bacteria. Because IFI16 has been reported to act as a sensor of various viral and bacterial DNAs^11–19^, we assumed that IFI16 also acts as a sensor protein for cytosolic dsDNA of mitochondrial origin. One day after the introduction of mitochondrial DNA into SH-SY5Y cells expressing flag-tagged IFI16 (IFI16-flag), the cytosolic fraction was collected and subjected to immunoprecipitation using an anti-dsDNA antibody. IFI16 was not precipitated in the control sample but was precipitated in the sample in which the intracellular introduction of mitochondrial DNA was performed (Fig. 3a and Supplementary Fig. 3a). IFI16 was also precipitated in the cytosolic fraction of PINK1-, ATP13A2- and GBA-depleted SH-SY5Y cells (Fig. 3a and Supplementary Fig. 3a). Immunofluorescence staining showed that cytosolic dsDNA colocalized with IFI16 in SH-SY5Y cells depleted of PINK1, ATP13A2, and GBA (Fig. 3b). When Alexa Fluor 594-conjugated mitochondrial DNA was introduced into SH-SY5Y cells, this exogenous DNA also clearly colocalized with IFI16 (Fig. 3c). We found that the downregulation of endogenous IFI16 in SH-SY5Y cells was difficult to achieve; thus, we used HeLa cells to determine the effect of endogenous IFI16 depletion. The downregulation or loss of endogenous IFI16 resulted in decreases in cell death and type I IFN responses in GBA- or ATP13A2-depleted HeLa cells (Fig. 3d–g and Supplementary Fig. 3b–d). Furthermore, overexpression of IFI16 in the cytosol reversed the effect of endogenous IFI16 depletion (Fig. 3h). These results suggested that IFI16 acts as a sensor of cytosolic dsDNA of mitochondrial origin.

### DNase II can rescue neurodegeneration in gba mutant zebrafish

**Fig. 4:**
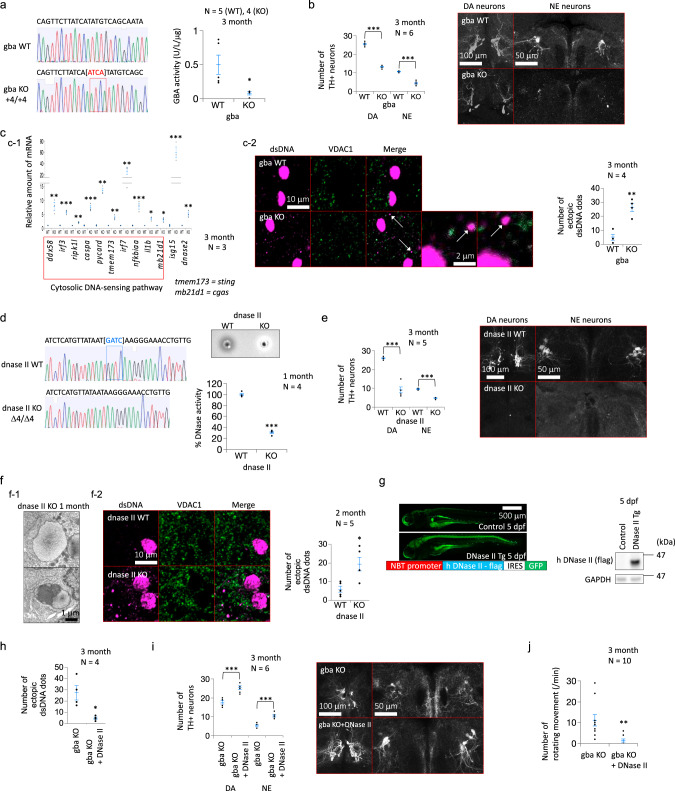
Effect of DNase II on neurodegeneration in gba KO zebrafish. **a** Loss of gba enzyme activity in gba KO zebrafish. WT: Wild type. KO: Knockout. U: unit. **b** Degeneration of tyrosine hydroxylase (TH) + neurons in gba KO zebrafish. The numbers of dopaminergic neurons (DA) in the posterior tuberculum (DC2 and DC4) and noradrenergic neurons (NE) in the locus coeruleus were significantly decreased at 3 months. WT: Wild type. KO: Knockout. **c** (**c-1**) RNA sequencing analysis in gba KO zebrafish at 3 months. WT: Wild type. KO: Knockout. **c-2** Cytosolic dsDNA deposits in gba KO zebrafish at 3 months. WT: Wild type. KO: Knockout. **d** Loss of DNase II enzyme activity in DNase II KO zebrafish at 1 month. WT: Wild type. KO: Knockout. **e** Degeneration of TH + neurons in DNase II KO zebrafish. The numbers of dopaminergic neurons in the posterior tuberculum (DC2 and DC4) and noradrenergic neurons in the locus coeruleus were significantly decreased at 3 months. TH: Tyrosine hydroxylase. DA: Dopaminergic neurons. NE: noradrenergic neurons. **f** (**f-1**) Transmission electron micrographs of DNase II KO zebrafish brain. KO: Knockout. **f-2** Cytosolic dsDNA deposits in DNase II KO zebrafish. WT: Wild type. KO: Knockout. **g** Details of NBT: human DNase II transgenic (Tg) zebrafish. NBT: *Xenopus* neural-specific beta-tubulin. h DNase II: Human DNase II. IRES: Internal ribosome entry site. **h** Cytosolic dsDNA deposits in gba KO zebrafish with or without human DNase II overexpression. KO: Knockout. **i** Number of tyrosine hydroxylase (TH) + neurons in gba KO zebrafish with or without human DNase II overexpression. KO: Knockout. DA: Dopaminergic neurons. NE: noradrenergic neurons. **j** Rotating movement in gba KO zebrafish with or without human DNase II overexpression. KO: Knockout. The statistical details are described in Supplementary Table 4. Source data of Fig. 4 are provided as a Source Data file.

Heterozygous mutations in GBA confer a high risk of sporadic PD, and our group and other researchers have previously reported that gba mutant fish show PD-like phenotypes, including movement disorders and the loss of dopaminergic neurons^20,21^. We generated a new strain of gba mutant zebrafish using TALEN-mediated genome editing to further analyze the relationship between the cytosolic dsDNA of mitochondrial origin and cell death in PD, and the homozygous mutants showed a decrease in the levels and activity of the gba protein in the brain (Fig. 4a and Supplementary Fig. 4a, b). These mutants exhibited reduced numbers of dopaminergic and noradrenergic neurons at 3 months (Fig. 4b). Transcripts involved in the cytosolic DNA-sensing pathway, including tmem173 (=sting) and mb21d1 (=cgas), were upregulated, and the number of cytosolic dsDNA puncta was increased in the brains of homozygous mutants (Fig. 4c). We next generated DNase II mutant zebrafish using TALEN-mediated genome editing, and the homozygous mutants showed reduced DNase activity under acidic conditions (Fig. 4d and Supplementary Fig. 4c, d). Although DNase II KO mice are embryonic lethal^22^, the homozygous mutant zebrafish were viable. This discrepancy probably occurred because DNase II is required for the enucleation of the erythroblast^22^, and zebrafish do not experience this phenomenon. Similar to the gba mutant zebrafish, the homozygous DNase II mutant zebrafish exhibited decreased numbers of dopaminergic and noradrenergic neurons and contained cytosolic DNA deposits (Fig. 4e, f). We crossed gba mutant zebrafish with a human DNase II transgenic line to investigate whether the removal of cytosolic dsDNA deposits ameliorates neurodegeneration in gba mutant zebrafish (Fig. 4g and Supplementary Fig. 4e). Indeed, the transgenic overexpression of human DNase II decreased cytosolic dsDNA deposits, rescued neurodegeneration, extended the life span, and improved the movement disorders of the homozygous gba mutants (Fig. 4h–j and Supplementary Fig. 4f, g; Supplementary Movies 1, 2). Collectively, the results showed that the cytosolic dsDNA of mitochondrial origin induced cell death and type I IFN responses in zebrafish in vivo. The reduction of these dsDNA deposits ameliorated the neurodegenerative phenotypes of the gba mutant zebrafish.

### Cytosolic dsDNA of mitochondrial origin and IFI16 accumulate in the brain of humans with PD

**Fig. 5:**
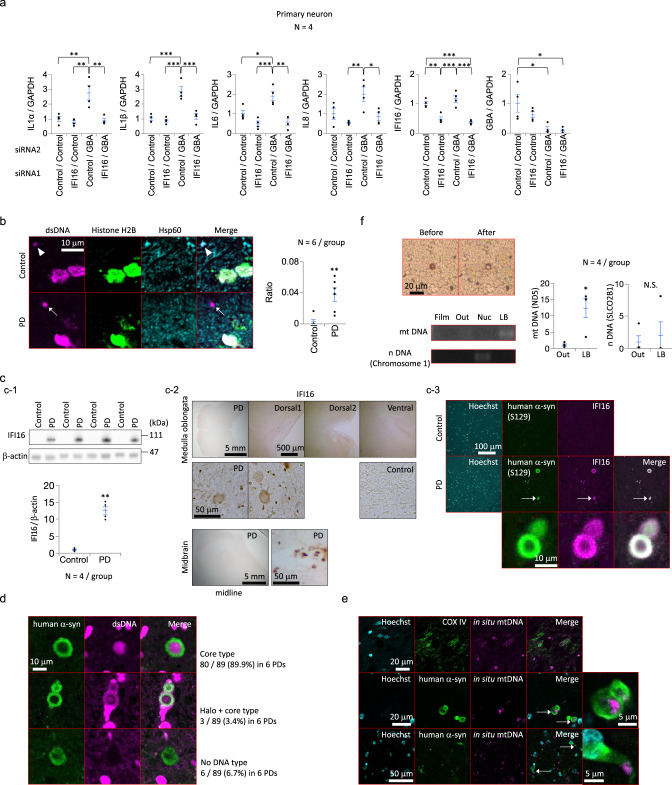
Accumulation of cytosolic dsDNA of mitochondrial origin in the brains of humans with Parkinson’s disease. **a** Effect of IFI16 depletion (siRNA knockdown) on type I IFN responses in human primary neurons following GBA knockdown. qPCR results for *IL-1α, IL-1β, IL-6, IL-8, IFI16,* and *GBA* mRNAs are shown. **b** Cytosolic dsDNA deposits in the brains of humans with PD (medulla oblongata). A white arrow indicates cytosolic dsDNA of mitochondrial origin. A white arrowhead indicates mtDNA within mitochondria. The graph shows the ratio of ectopic dsDNA+ number/Hoechst 33258+ cell number in the brain specimens. PD: Parkinson’s disease. **c** (**c-1**) Western blot of the IFI16 protein in brains (amygdala) of humans with PD. PD: Parkinson’s disease. **c-2** Immunohistochemistry of the IFI16 protein in brains (medulla oblongata and midbrain) of humans with PD. Note that PD pathology is often observed in the amygdala and dorsal nucleus of the vagus nerve in the medulla oblongata^43,44^. PD: Parkinson’s disease. **c-3** Immunofluorescence of the IFI16 protein in brains (medulla oblongata) of humans with PD. The white arrow indicates a Lewy body containing α-synuclein and IFI16. PD: Parkinson’s disease. α-syn: α-synuclein. **d** Immunofluorescence staining of human brain tissues using an anti-dsDNA antibody and anti-α-synuclein antibody. α-syn: α-synuclein. **e** In situ hybridization of human brain tissues (medulla oblongata) using mitochondrial DNA probes. White arrows indicate Lewy bodies or Lewy neurites containing mitochondrial DNA. α-syn: α-synuclein. **f** Laser microdissection of brain sections (medulla oblongata) and subsequent PCR amplification of mitochondrial or nuclear DNA sequences. The graph shows the relative amount (qPCR) of mitochondrial or nuclear DNA. “Before” shows DAB staining performed just before laser microdissection, and “After” shows staining after microdissection. Film: samples dissected from only the cover film, Out: samples dissected from areas without Lewy bodies in the brain specimens, Nuc: samples dissected from the nuclei in the brain specimens (HE staining), LB: samples dissected from the cores of Lewy bodies in the brain specimens. mtDNA: Mitochondrial DNA. n NDA: Nuclear DNA. N.S.: statistically not significant. The statistical details are described in Supplementary Table 4. Source data of Fig. 5 are provided as a Source Data file.

The downregulation of endogenous IFI16 resulted in the reduction of type I IFN responses in GBA-depleted human primary neurons (Fig. 5a). Hence, we inspected postmortem human brain tissues from patients who were definitively diagnosed with idiopathic PD by pathological examinations. The abundance of cytosolic dsDNA of mitochondrial origin was increased in the medulla oblongata of postmortem brain tissues from patients with PD (Fig. 5b). The levels of the IFI16 protein were markedly increased in these brain tissues from PD patients (Fig. 5c and Supplementary Fig. 5a). Lewy bodies are abnormal aggregates of the α-synuclein protein detected in the brains of patients with PD. Surprisingly, the IFI16 deposits clearly colocalized with the halos of these Lewy bodies in the medulla oblongata of patients with PD (Fig. 5c). dsDNA deposits were also observed in Lewy bodies from patients with PD (Fig. 5d–f and Supplementary Fig. 5b–d). Altogether, the results demonstrated that cytosolic dsDNA of mitochondrial origin accumulated in PD brains and that these dsDNA deposits and IFI16 might play important roles in human PD pathogenesis.

## Discussion

In the present study, the cytosolic content of mitochondrial DNA was shown to be aberrantly increased in PD models, leading to type I IFN responses and cell death, ultimately resulting in neurodegeneration. Reducing the abundance of cytosolic dsDNA of mitochondrial origin can ameliorate cell death and neurodegeneration in these PD models. Furthermore, the levels of cytosolic dsDNA of mitochondrial origin and its sensor protein, IFI16, were found to be increased in postmortem brain tissues from PD patients. Based on these results, we propose that the cytosolic leakage of mitochondrial DNA is highly toxic and could be a common and important characteristic of familial and idiopathic PD.

If the mitochondria are damaged and/or autophagy–lysosome functions are impaired, mitochondrial DNA, which is typically rapidly degraded, persists in the cytosol and induces cytotoxicity (Supplementary Fig. 5e). Consistent with our model, the loss of STING was recently shown to prevent inflammation, motor defects and neurodegeneration in Prkn^−/−^;mutator mice^23^. The suppression of the cGAS-STING pathway can decrease inflammation but may also increase the formation of malignant tumors^24,25^. Due to the lack of a definitive IFI16 ortholog in animal models, most of the studies related to IFI16, including our own, have been conducted via cell-based experiments. IFI16 has been reported to act as a sensor of various viral and bacterial DNAs^11–19^. Our experiments show that IFI16 also serves as a sensor of cytosolic dsDNA of mitochondrial origin and mediates type I IFN responses and cell death. Strategies that increase DNase II activity and/or inhibit IFI16 activity could represent a potent therapeutic approach for PD.

Several questions have emerged from this work. For example, many reports have shown that the cytosolic leakage of nuclear DNA is sensed by cGAS^26,27^. Hence, what is the difference between the leakage of nuclear DNA and mitochondrial DNA? Not only the origin of DNA but also the forms of DNA should be also important. There will be single strand or dsDNA, linear or circular DNA, short or long DNA, with some proteins including histone or without, aggregated or soluble DNA and some show higher-order structure. The difference between the sensors and downstream reactions should also be addressed. In addition to cGAS and IFI16, other sensor molecules of nucleic acids have been identified, including DDX41^28^, TLR9^29^, AIM2^30,31^, HMGBs^32^, DHX36, DHX9^33^, ZBP1^34^, or MRE11^35^. Thus, what is the division of roles among these sensor molecules? The differences and cross-talk among these sensor molecules should also be clarified. The mechanism of cytosolic leakage of mitochondrial DNA is unknown. Mitochondrial DNA seemed to leak out of the mitochondria through Tom20 positive mitochondrial membrane. In addition to passive leakage of the mitochondrial content, there might be an active release of the mitochondrial DNA. Kim et al. showed mitochondrial DNA is released via VDAC pores under oxidative stress^36^. There can be another mechanism or multiple mechanisms can co-exist. The mechanism of mitochondrial DNA leakage should be addressed in the near future. We analyzed human skin fibroblast from control, PD patients, or Gaucher’s disease patients. Although ectopic dsDNA dots were increased in the fibroblasts derived from PD or Gaucher’s disease patients, the amount of IL6 mRNA was comparable among control, PD, and Gaucher’s disease group (Supplementary Fig. 5f and Supplementary Table 1). The reason for this negative finding is unknown, but the fibroblast can adapt to the chronic disease condition. Finally, in this paper, we concentrate on the role of cytosolic dsDNA of mitochondrial origin in PD, and it is of particular interest whether cytosolic dsDNA of mitochondrial origin can induce other diseases in different organs.

## Methods

### Ethics statement

All animal experiments were performed in compliance with the protocol reviewed by the Institutional Animal Care and Use Committee and approved by the President of Niigata University (Permit Number: #28 Niigata Univ. Res.367-1). Informed consent was obtained from the participants of human studies. The study using postmortem brain samples from human subjects was approved by the Ethical Review Boards of Niigata University (#2515). Patient profiles are described in Supplementary Table 2.

### Cell lines

SH-SY5Y cells were subcloned from SK-N-SH cells, which were isolated from a bone marrow biopsy from a female with neuroblastoma (EC94030304-F0, DS Pharma Biomedical, Suita, Japan). SH-SY5Y cells were cultured in DMEM/Ham’s F-12 (Nacalai Tesque, Kyoto, Japan) supplemented with 10% fetal bovine serum (FBS, BioWest, Nuaillé, France) and 1% penicillin–streptomycin solution (Wako) at 37 °C under 5% CO_2_. HeLa cells were derived from cervical cancer cells from a female (ATCC, CCL-2). HeLa cells were cultured in DMEM (Nacalai Tesque) supplemented with 10% FBS (Biowest) and a 1% penicillin–streptomycin solution (Wako) at 37 °C under 5% CO_2_. HEK293 cells were derived from kidneys of a fetus and HEK293T cells are expressing SV40 large T antigen (RIKEN cell bank, Tsukuba, Japan). HEK293T cells were cultured in DMEM (Nacalai Tesque) supplemented with 10% FBS (Biowest) and a 1% penicillin–streptomycin solution (Wako) at 37 °C under 5% CO_2_. Human primary neurons (ScienCell Research Laboratories, Carlsbad, CA, USA) were grown in a neuronal medium (ScienCell Research Laboratories) at 37 °C in a 5% CO_2_ atmosphere. IFI16-deficient HeLa cells were generated using the CRISPR–Cas9 system. The IFI16 gRNA sequence was cloned into pSpCas9 (BB)-2A-Puro (PX495) v.2.0 (plasmid 62988; Addgene, Cambridge, MA, USA) using the method described by Ran et al.^37^. The overexpression of the plasmid DNA in HeLa cells was performed using a NEPA21 electroporator (Nepagene; poring pulse: 125 V; 7.5 ms; 50 ms interval; 2 pulses; 10% decay rate; + polarity, transfer pulse: 20 V; 50 ms; 50 ms interval; 5 pulses; 40% decay rate; +/− polarity). One day after electroporation, the cells were diluted in 10-cm plates. After successful colony formation, the cells were cloned using cloning rings. Dilution and colony formation for cloning were performed twice. The depletion of IFI16 in the stable cell line was verified and monitored using Western blotting and DNA sequencing.

### Fish strains and maintenance

The zebrafish AB strain (https://zfin.org/ZDB-GENO-960809-7) was used in this study. Zebrafish were raised and maintained under a 14-h light/10-h dark cycle at 28 °C using standard protocols. The fish were fed brine shrimp at 9:00 a.m. and powdered feed (Kyorin, Himeji, Japan) at 12:00 p.m. Feeding began 5 days postfertilization. The generation of mutant and transgenic zebrafish was described elsewhere.

### RNA isolation and qRT-PCR

Total cellular RNA was isolated using TRIzol reagent according to the manufacturer’s instructions (ThermoFisher Scientific, Waltham, MA, USA). The cDNA templates were synthesized from the purified RNA using the PrimeScript 1st strand cDNA Synthesis Kit (Takara Bio, Kusatsu, Japan) with oligo (dT)_20_ primers. qPCR was performed using TB Green Premix Ex Taq II (Takara Bio) and analyzed in a Thermal Cycler Dice Real Time System Lite (Takara Bio). The PCR primers used in the present study are listed in Supplementary Table 3.

### Fractionation of the cytosol and qPCR

The cytosolic fraction of HeLa cells was separated using a Subcellular Protein Fractionation Kit for Cultured Cells (ThermoFisher Scientific). qPCR was performed using TB Green Premix Ex Taq II (Takara Bio) and the Human Mitochondrial DNA (mtDNA) Monitoring Primer Set (Takara Bio) in a Thermal Cycler Dice Real Time System Lite (Takara Bio).

### siRNA and plasmid transfection and western blotting

The expression of genes of interest was silenced in SH-SY5Y cells using Accell siRNAs in Accell siRNA delivery media (Dharmacon, Lafayette, CO, USA) according to the manufacturer’s instructions. Accell siRNAs were used at a final concentration of 1 μM unless otherwise stated. For comparisons between single, double and triple siRNA treatments, total siRNAs were administered at a final concentration of 3 μM (for example, single PINK1 siRNA = 1 μM PINK1 siRNA + 2 μM control siRNA, and triple siRNA = 1 μM PINK1 siRNA + 1 μM ATP13A2 siRNA + 1 μM GBA siRNA). The expression of genes of interest was silenced in HeLa cells using Stealth RNAi siRNA (ThermoFisher Scientific) in Lipofectamine RNAiMAX Transfection Reagent (ThermoFisher Scientific) according to the manufacturer’s instructions. The expression of genes of interest was silenced in human primary neurons using Stealth RNAi siRNA (ThermoFisher Scientific) in the Altofect Transfection Reagent (Altogen Biosystems, Las Vegas, NV, USA) according to the manufacturer’s instructions. Cells were harvested 3 days or 4 days after siRNA transfection in SH-SY5Y or HeLa cells, respectively, unless otherwise stated. siRNAs were transfected 7 days after the initial seeding of human primary neurons, and neurons were harvested 4 days after siRNA transfection. The siRNAs used in the present study are listed in Supplementary Table 3. The overexpression of plasmid DNA in SH-SY5Y cells was performed using a NEPA21 electroporator (Nepagene, Chiba, Japan; Poring pulse: 175 V; 2.5 ms; 50 ms interval; 2 pulses; 10% decay rate; + polarity, transfer pulse: 20 V; 50 ms; 50 ms interval; 5 pulses; 40% decay rate; +/− polarity). The introduction of mitochondrial DNA in SH-SY5Y cells was performed using a NEPA21 electroporator with the same parameters. The overexpression of plasmid DNA in HeLa cells was performed using a NEPA21 electroporator (Nepagene; Poring pulse: 125 V; 7.5 ms; 50 ms interval; 2 pulses; 10% decay rate; + polarity, transfer pulse: 20 V; 50 ms; 50 ms interval; 5 pulses; 40% decay rate; +/− polarity).

For western blotting, total protein extracts were prepared in lysis solution (50 mM Tris, pH 8.0, 150 mM NaCl, 1% (v/v) NP-40, 0.5% (w/v) deoxycholate, and 0.1% (w/v) SDS). Protein concentrations were determined using the BCA protein assay (ThermoFisher Scientific). Protein extracts were separated in SDS-PAGE gels and transferred to a PVDF membrane (GE Healthcare, Chicago, IL, USA). Western blotting was performed according to standard protocols (Dilution of the 1st antibody: 1/2500). The details of the antibodies used in the present study are listed in Supplementary Table 3.

Cell death was measured using propidium iodide (PI) staining (Dojindo, Kumamoto, Japan), an LDH release assay (LDH Cytotoxicity Detection Kit, Takara Bio) or caspase-3 immunostaining (1/100, Abcam, Cambridge, UK). The cell culture medium was exchanged 24 h before the LDH release assay. Cell viability was measured using a WST-8 assay (Cell Counting Kit-8, Dojindo).

### Generation of mutant and transgenic zebrafish

Zebrafish mutants were generated using the TALEN method. The TALEN target sites in gba and DNase II were designed at the TAL Effector Nucleotide Targeter (TALE-NT) website (https://tale-nt.cac.cornell.edu/)^38^. TALEN plasmids were assembled with the Golden Gate TALEN and TAL Effector kit 2.0^39^ according to the protocol published previously by Addgene (Cambridge, MA, USA) with slight modifications. Repeat variable di-residue (RVD) modules were cloned into pCS2TAL3DD and pCS2TAL3RR to generate the left TALEN and right TALEN, respectively. The TALEN sites were TGCTGCCAGATGCTGGTCAGTTCTTATCATATGTCAGCAATAAAGCTGGCAGCA for gba and TTTTCCTCCATCTCATGTTATAATGATCAAGGGAAACCTGTTGACTGGTTTGTA for DNase II (spacer sequence underlined). The capped mRNAs for the left and right TALENs were generated from *Not*I-digested pCS2TAL3DD and pCS2TAL3RR plasmids using the mMESSAGE mMACHINE SP6 kit (ThermoFisher Scientific). Zebrafish embryos were microinjected at the one-cell stage. Injected founders (F0s) were grown to adulthood and outcrossed to wild-type AB partners, and genomic DNA was extracted from individual F1 embryos for PCR amplification and direct sequencing to identify the germline transmission of the mutations. The F1 generation and subsequent generations were genotyped using PCR (gba: forward primer: CGGAATAATCACCACAGCAA, reverse primer: AAGAGCACTCACCTGCACCT, DNase II: forward primer: GCGGATTTCCATCATGTTTC, reverse primer: GGCTCACATTGTCCTTTTAGG) and direct sequencing (gba: CGGAATAATCACCACAGCAA, DNase II: GCGGATTTCCATCATGTTTC). Heterozygous mutant fish were crossed to obtain homozygous mutant (gba KO or DNase II KO) and control fish. The oligos used for PCR and sequencing are listed in Supplementary Table 3.

Plasmid DNA (*Xenopus* neural-specific beta-tubulin (NBT) promoter: human DNase II-flag IRES GFP) was microinjected into the soma of one-cell stage wild-type embryos (=P0 generation, mosaic) at a concentration of 25 ng/ml to generate human DNase II transgenic fish. For the generation of stable transgenic fish, transgene cassettes flanked by recognition sites for the Tol2 transposase were used, and the DNA was supplemented with 25 ng/ml mRNA encoding the Tol2 transposase to increase the genomic integration of the transgene cassette^40^. The injected P0 generation was raised to adulthood and crossed with wild-type fish to screen embryos of the F1 generation for the inherited expression of the fluorescent protein under a fluorescence stereomicroscope (MZ10F, Leica Microsystems, Wetzlar, Germany).

### Measurement of GBA and DNase II activities

GBA activity was determined using a beta-glucosidase assay kit (Abnova, Taipei, Taiwan). The kit uses *p*-nitrophenyl-β-d-glucopyranoside (β-NPG), which is specifically hydrolyzed by β-glucosidase to generate a yellow product. Zebrafish brains were homogenized in 10 volumes of ice-cold TBS buffer (50 mM Tris-HCl and 150 mM NaCl, pH 7.4). The homogenate was clarified via centrifugation at 1000×*g* for 5 min at 4 °C. The supernatant was subjected to ultracentrifugation at 100,000×*g* for 1 h at 4 °C using an Optima MAX-XP (Beckman Coulter, Brea, CA, USA). The supernatant was used as the TBS-soluble fraction. Samples (4 μg) of the TBS-soluble fraction of the brain homogenate were placed in 96-well plates and incubated with β-NPG (final concentration: 1.0 mM) at RT. The OD was measured at 405 nm at the beginning of the experiment (*t* = 0) and after 15 h (*t* = 15 h) using a plate reader (SpectraMax 250, Molecular Devices, San Jose, CA, USA).

DNase II activity was determined using the single radial enzyme-diffusion (SRED) method^41^. Zebrafish brains were prepared in lysis solution (50 mM Tris, pH 8.0, 150 mM NaCl, 1% (v/v) NP-40, 0.5% (w/v) deoxycholate, and 0.1% (w/v) SDS). Brain homogenates with 1 μg of protein/5 μl were placed in cylindrical wells (radius, 1.5 mm) punched into a 2% (w/v) agarose gel containing 0.1 mg/ml zebrafish genomic DNA, 1/10,000 SYBR Green I Nucleic Acid Gel Stain (Takara Bio), 0.1 M sodium acetate buffer (pH 4.7) and 20 mM EDTA. After incubation at 37 °C for 18 h, the radius of the dark circle was measured under a UV transilluminator at 365 nm. DNase II activity related to mitochondrial DNA degradation was also assessed as follows. Brain homogenates (5 μg protein) were mixed with 1 μg of zebrafish mitochondrial DNA in 0.1 M sodium acetate buffer (pH 4.7) and 20 mM EDTA, followed by incubation at 37 °C for 5 min. The degradation of mitochondrial DNA was assessed using agarose gel electrophoresis.

### Immunofluorescence Labeling

For the imaging and counting of cytosolic dsDNA deposits, cells were washed twice with PBS and fixed with ice-cold methanol for 10 min at 4 °C. The cells were washed three times with PBST for 5 min each, then permeabilized with PBS containing 0.2% (w/v) Triton X-100 for 10 min. The cells were washed three times with PBST for 5 min each and incubated with 2% (w/v) BSA in PBST for 30 min. The cells were incubated with a primary antibody against H2B (1/400, Abcam) diluted in 2% (w/v) BSA in PBST for 1 h at RT. The cells were washed three times with PBST for 5 min each and incubated with an Alexa Fluor 488-conjugated AffiniPure donkey anti-chicken IgY secondary antibody (IgG) (H + L) (1/200, Wako) diluted in 2% (w/v) BSA in PBST for 1 h at RT. The cells were washed three times for 5 min each with PBST. Primary antibodies against dsDNA (1/800, Abcam) and Hsp60 (1/400, Abcam) diluted in 2% (w/v) BSA in PBST were applied for 1 h at RT. The cells were washed three times with PBST for 5 min each then incubated with highly cross-adsorbed Alexa Fluor 680-conjugated donkey anti-rabbit IgG (H + L) (1/200, ThermoFisher Scientific) and Alexa Fluor 594-conjugated donkey anti-mouse IgG (H + L) secondary antibodies (1/200, ThermoFisher Scientific) diluted in 2% (w/v) BSA in PBST for 1 h at RT. The cells were washed three times with PBST for 5 min each. Images of Hsp60 staining (=mitochondrial matrix) were subtracted from images of dsDNA staining, and dsDNA puncta ranging in size from 5 – 20 μm^2^ were selected using ImageJ software (National Institutes of Health, Bethesda, MD, USA). The dsDNA puncta that colocalized with H2B were eliminated, and the number of cells containing dsDNA puncta without an Hsp60/H2B signal was counted.

For the subcellular localization of dsDNA, cells were washed twice with PBS for 5 min each and fixed with 4% (w/v) paraformaldehyde in phosphate buffer solution (Nacalai) for 10 min. The cells were washed three times with PBST for 5 min each then permeabilized with PBS containing 0.2% (w/v) Triton X-100 for 10 min. The cells were washed three times with PBST for 5 min each and incubated with 2% (w/v) BSA in PBST for 30 min. Primary antibodies against dsDNA (1/800, Abcam), LC-3 (1/400, Medical and Biological Laboratories, Nagoya, Japan), Tom20 (1/400, Cell Signaling Technology, Danvers, MA, USA), α-synuclein (1/400, Novus Biologicals, Littleton, CO, USA), Flag (1/400, ab1170, Abcam), Flag (1/400, F1804, Sigma-Aldrich) or cathepsin D (1/400, Merck Millipore, Burlington, MA, USA) were diluted in 2% (w/v) BSA in PBST, and the cells were incubated with the antibodies for 1 h at RT. The cells were washed three times with PBST for 5 min each, and the corresponding secondary antibodies (listed in Supplementary Table 3) diluted in 2% (w/v) BSA in PBST were applied to the cells. The cells were incubated for 1 h at RT and washed three times with PBST for 5 min each. For the triple staining of Hsp60, LC3, and dsDNA, or α-synuclein, cathepsin D and dsDNA, Picogreen (1/10000, Quant-iT PicoGreen dsDNA Assay Kit, ThermoFisher Scientific) was used instead of the dsDNA antibody.

For the imaging and counting of cytosolic dsDNA deposits in zebrafish sections, dewaxed paraffin sections (10 μm thickness) of the diencephalon in the axial orientation were washed three times with PBS for 5 min each and incubated with 2% (w/v) BSA in PBS for 30 min. Primary antibodies against dsDNA (1/800, Abcam) and VDAC1/porin (1/400, Abcam) diluted in 2% (w/v) BSA in PBS were incubated with the sections for 1 h at RT. The sections were washed three times with PBS for 5 min each. The following secondary antibodies were diluted in 2% (w/v) BSA in PBS: an Alexa Fluor 488-conjugated goat anti-rabbit IgG (H + L) cross-adsorbed secondary antibody (1/200, ThermoFisher Scientific) and an Alexa Fluor 594-conjugated goat anti-mouse IgG (H + L) cross-adsorbed secondary antibody (1/200, ThermoFisher Scientific). The sections were incubated with the secondary antibodies for 1 h at RT and washed three times with PBS for 5 min each. Images of VDAC staining (=mitochondria) were subtracted from images of dsDNA staining, and dsDNA puncta ranging in size from 0.2 to 2 μm^2^ were counted in a 100-μm square field using ImageJ software (National Institutes of Health).

For the imaging of cytosolic dsDNA in postmortem brain tissues from human subjects, dewaxed paraffin sections (5 μm thickness) of the medulla oblongata cut in the axial orientation were incubated in 10 mM sodium citrate buffer (pH 6.0) at 121 °C for 15 min in an autoclave (HA-300MIV, HIRAYAMA, Kasukabe, Japan). The sections were cooled at RT then incubated in methanol overnight at 4 °C. The sections were washed three times with PBS for 5 min each and incubated with 2% (w/v) BSA in PBS for 30 min. The sections were incubated with a primary antibody against H2B (1/400, Abcam) diluted in 2% (w/v) BSA in PBS for 1 h at RT. The sections were washed three times with PBS for 5 min each and incubated with an Alexa Fluor 488-conjugated AffiniPure donkey anti-chicken IgY secondary antibody (IgG) (H + L) (1/200, Wako) diluted in 2% (w/v) BSA in PBS for 1 h at RT. The sections were washed three times for 5 min each with PBS. Primary antibodies against dsDNA (1/800, Abcam) and Hsp60 (1/400, Abcam) diluted in 2% (w/v) BSA in PBS were applied for 1 h at RT. The sections were washed three times with PBS for 5 min each, and highly cross-adsorbed Alexa Fluor 680-conjugated donkey anti-rabbit IgG (H + L) (1/200, ThermoFisher Scientific) and Alexa Fluor 594-conjugated donkey anti-mouse IgG (H + L) secondary antibodies (1/200, ThermoFisher Scientific), diluted in 2% (w/v) BSA in PBS, were applied to the cells for 1 h at RT. The sections were washed three times with PBS for 5 min each and mounted with the SlowFade Gold antifade reagent (ThermoFisher Scientific). Images of Hsp60 staining were subtracted from images of dsDNA staining, and dsDNA puncta ranging in size from 1 to 5 μm^2^ were selected using ImageJ software (National Institutes of Health). The dsDNA puncta that colocalized with H2B were eliminated, and the number of cells containing dsDNA puncta without an Hsp60/H2B signal was counted.

For the imaging of dsDNA and Lewy bodies in postmortem brain tissues from human subjects, dewaxed paraffin sections (5 μm thickness) of the medulla oblongata cut in the axial orientation were washed three times with PBS for 5 min each and incubated with 2% (w/v) BSA in PBS for 30 min. Primary antibodies against dsDNA (1/800, Abcam) and α-synuclein (1/200, Abcam) or phosphorylated α-synuclein (1/200, Wako) diluted in 2% (w/v) BSA in PBS were applied to the sections for 1 h at RT. The sections were washed three times with PBS for 5 min each. The following secondary antibodies were diluted in 2% (w/v) BSA in PBS: Alexa Fluor 488-conjugated goat anti-rabbit IgG (H + L) cross-adsorbed secondary antibody (1/200, ThermoFisher Scientific) and Alexa Fluor 594-conjugated goat anti-mouse IgG (H + L) cross-adsorbed secondary antibody (1/200, ThermoFisher Scientific). The sections were incubated with the secondary antibodies for 1 h at RT and washed three times with PBS for 5 min each.

For the imaging of IFI16 in postmortem brain tissues from human subjects, dewaxed paraffin sections (5 μm thickness) of the medulla oblongata cut in the axial orientation were incubated in 10 mM sodium citrate buffer (pH 6.0) at 121 °C for 15 min in an autoclave. The sections were cooled at RT, washed three times with PBS for 5 min each, and incubated with 2% (w/v) BSA in PBS for 30 min. Primary antibodies against IFI16 (1/200, Abcam) and α-synuclein (1/200, Abcam) diluted in 2% (w/v) BSA in PBS were applied to the sections for 1 h at RT. The sections were washed three times with PBS for 5 min each, and an Alexa Fluor 488-conjugated goat anti-rabbit IgG (H + L) secondary antibody (1/100, ThermoFisher Scientific), an Alexa Fluor 594-conjugated goat anti-mouse IgG (H + L) secondary antibody (1/100, ThermoFisher Scientific) and Hoechst 33258 (10 μg/ml, Dojindo) diluted in 2% (w/v) BSA in PBS were applied to the cells for 1 h at RT. The sections were washed three times with PBS for 5 min each and mounted with the SlowFade Gold antifade reagent (ThermoFisher Scientific).

Immunofluorescence images were obtained using an A1R + confocal microscope (Nikon, Tokyo, Japan). The details of the antibodies used in the present study are listed in Supplementary Table 3.

### Immunohistochemistry

For DAB staining of IFI16 in postmortem brain tissues from human subjects, dewaxed paraffin sections (5 μm thickness) of the medulla oblongata cut in the axial orientation were incubated in 10 mM sodium citrate buffer (pH 6.0) at 121 °C for 15 min in an autoclave. The sections were cooled at RT, washed three times with PBS for 5 min each, and incubated with 2% (w/v) BSA in PBS for 30 min. A primary antibody against IFI16 (1/500, Abcam) diluted in 2% (w/v) BSA in PBS was applied to the sections for 1 h at RT. The sections were washed three times with PBS for 5 min each and incubated with biotinylated goat anti-rabbit IgG (1/500, Vector Laboratories, Burlingame, CA, USA) diluted in 2% (w/v) BSA in PBS for 1 h at RT. The sections were washed three times with PBS for 5 min each. The sections used for DAB staining were further incubated with the VECTASTAIN Elite ABC HRP Kit reagent (Vector Laboratories) for 30 min, washed three times with PBS for 5 min each, developed with ImmPACT DAB Peroxidase Substrate (Vector Laboratories), and washed three times with PBS for 5 min each. After washing with distilled water, the sections were immersed in ethanol then xylene, mounted with Mount Quick (Daido Sangyo, Toda, Japan), and analyzed using an upright microscope (Leica Microsystems DM2500LED). The details of the antibodies used in the present study are listed in Supplementary Table 3.

### Immunoprecipitation

Protein G Sepharose beads (GE Healthcare) were washed and resuspended in PBS. Cells were harvested using a cell scraper and fixed for 10 min at RT in 1% formaldehyde. After fixation, 140 mM glycine was added, and the cells were incubated for 10 min at RT, then washed 3 times in ice-cold PBS. The cytosolic and nuclear fractions of SH-SY5Y cells were separated using a Subcellular Protein Fractionation Kit for Cultured Cells (ThermoFisher Scientific). For the immunoprecipitation of dsDNA in the cytosol, 5 µg of an anti-dsDNA antibody (Abcam) was added to the cytosolic fraction of SH-SY5Y cells. After 1 h of incubation at RT on a shaker, antibodies were bound to 40 µl of beads for 1 h at RT on a shaker. After washing three times in 1 ml of PBS, 100 µl of 2× SDS-PAGE sample buffer (80 mM Tris pH 6.8, 2.0% SDS, 10% glycerol, 0.0006% bromophenol blue, 0.1 M DTT) was added, followed by boiling at 95 °C for 10 min. Following centrifugation at 12,000×*g* at 4 °C for 5 min, the supernatant was subjected to SDS-PAGE and Western blotting.

### Electron microscopy

For zebrafish brain, samples were fixed with 2% PFA and 2% glutaraldehyde in 0.1 M cacodylate buffer, pH 7.4, overnight at 4 °C. After washing three times with 0.1 M cacodylate buffer, samples were fixed with 2% osmium tetroxide in 0.1 M cacodylate buffer at 4 °C for 2 h. After dehydration using graded ethanol solutions, the samples were infiltrated with propylene oxide (PO) twice for 30 min each and transferred to a 70:30 mixture of PO and resin (Quetol-812, Nisshin EM Co., Tokyo, Japan) for 1 h. The samples were transferred to fresh 100% resin and polymerized at 60 °C for 48 hr. For cell culture, samples were fixed with 2% PFA and 2% glutaraldehyde in 0.1 M phosphate buffer, pH 7.4, at 37 °C then placed in a refrigerator for 30 min. The samples were fixed in 2% glutaraldehyde in 0.1 M phosphate buffer overnight at 4 °C. The samples were washed three times with 0.1 M phosphate buffer for 30 min and postfixed with 2% osmium tetroxide in 0.1 M phosphate buffer at 4 °C for 1 h. The samples were dehydrated in graded ethanol solutions, transferred to resin (Quetol-812, Nisshin EM Co.), and polymerized at 60 °C for 48 h.

The polymerized resins were cut into ultrathin 70-nm sections with a diamond knife using an ultramicrotome (Ultracut UCT, Leica Microsystems) and mounted on copper grids. The sections were stained with 2% uranyl acetate at RT for 15 min and washed with distilled water, followed by secondary staining with a lead staining solution (Sigma-Aldrich) at RT for 3 min. The grids were observed under a transmission electron microscope (JEM-1400Plus, JEOL Ltd., Tokyo, Japan) at an acceleration voltage of 80 kV (zebrafish brain) or 100 kV (cell) and images were recorded using a CCD camera (EM-14830RUBY2, JEOL Ltd.).

### Propidium iodide staining

The overexpression of plasmid DNA (CMV promoter: GFP, or CMV promoter: human DNase II-flag IRES GFP) in SH-SY5Y cells was performed using a NEPA21 electroporator (Nepagene) (poring pulse: 175 V; 2.5 ms; 50 ms interval; 2 pulses; 10% decay rate; + polarity, transfer pulse: 20 V; 50 ms; 50 ms interval; 5 pulses; 40% decay rate; +/− polarity). These cells were passaged twice at 1 day and 2 days after electroporation to remove cell debris. The expression of genes of interest was silenced using Accell siRNA in Accell siRNA delivery media (Dharmacon) according to the manufacturer’s instructions 1 day after the final passage. The percentages of dead cells (GFP+PI+/GFP+) were determined by staining cultures with Hoechst 33258 (10 μg/ml, Dojindo) and PI (10 μg/ml, Dojindo) 3 days after siRNA treatment. Images were captured and counted using an A1R+ confocal microscope (Nikon).

### Immunostaining of TH+ neurons in Zebrafish

The immunostaining and counting of TH+ neurons in zebrafish were performed as previously described^42^. Briefly, adult fish were sacrificed by the addition of 0.1% tricaine to the circulating water of the breeding system. Brains were removed, explanted, and fixed with 4% (w/v) paraformaldehyde in PBS (Nacalai) at 4 °C O/N. The specimens were embedded in 2% low-melting agarose, and 200 μm axial sections were prepared using a PRO7 microslicer (Dosaka EM, Kyoto, Japan). Floating slices were incubated with 10 mM sodium citrate buffer, pH 8.5, at 80 °C for 120 min. After washing with PBS containing 1% Triton X-100, sections were blocked with 2% BSA in PBS/1% Triton X-100 buffer for 30 min. These pretreated sections were incubated with a rabbit anti-TH antibody (1/500, AB152, EMD-Millipore, Billerica, MA, USA) O/N at 4 °C. After washing with PBS/1% Triton X-100 buffer, the sections were incubated with anti-rabbit IgG conjugated to Alexa Fluor 594 (1/100, ThermoFisher Scientific) O/N at 4 °C. The sections were washed with PBS/1% Triton X-100 buffer and analyzed using an A1R+ confocal microscope (Nikon).

### In situ hybridization of mitochondrial or nuclear DNA

Human mitochondrial DNA was isolated from HEK293T cells using the mtDNA Extractor CT Kit (Wako). Human nuclear DNA was isolated from HEK293T cells using the NE-PER Nuclear and Cytoplasmic Extraction Reagents (ThermoFisher Scientific) and subsequent DNA extraction. Probes were produced using traditional nick translation protocols and a FISH Tag DNA Red Kit with Alexa Fluor 594 dye (ThermoFisher Scientific). DNA hybridization was performed according to the manufacturer’s protocol. Briefly, dewaxed paraffin slides (3 μm thickness) or cells fixed with 4% (w/v) paraformaldehyde in PBS (Nacalai) were incubated with 2× SSC for 2 min at 73 °C then incubated with 0.05% (w/v) pepsin and 10 mM HCl for 10 min at 37 °C. After washing the specimens twice with PBS for 5 min each, the specimens were treated with 1% (w/v) formaldehyde in PBS for 5 min at RT. After two washes with PBS for 5 min each, the specimens were dehydrated in 70%, 85%, and 100% ethanol for 1 min each at RT. After air drying, the specimens were incubated with 70% (v/v) formamide/2× SSC (pH 7.0) at 72 °C for 2 min. The specimens were dehydrated in 70%, 80%, and 95% cold ethanol (–20 °C) for 2 min each and air-dried at RT. Next, 2.5 μl of the DNA probe (4 ng/μl), 1 μl of 20X SSC, and 6.5 μl of formamide were mixed, denatured at 72 °C for 5 min and cooled on ice. The probes were applied to the samples, which were then sealed with coverslips and incubated in a humidified chamber at 37 °C O/N. The specimens were soaked in 2X SSC/0.1% (v/v) NP-40 at 37 °C for 5 min, and the coverslips were removed. The specimens were incubated with 0.4X SSC/0.3% (v/v) NP-40 at 73 °C for 2 min then with 2× SSC/0.1% (v/v) NP-40 at RT for 1 min. The specimens were washed three times with PBS for 5 min each and incubated with 2% (w/v) BSA in PBS for 30 min. For human brain sections, a primary antibody against α-synuclein (1/200, Abcam) or cytochrome *c* oxidase subunit IV (COX IV, 1/200, Abcam) was diluted in 2% (w/v) BSA in PBS then applied to the sections for 1 h at RT. The sections were washed three times with PBS for 5 min each then incubated with an Alexa Fluor 488-conjugated goat anti-rabbit or goat anti-mouse IgG (H + L) cross-adsorbed secondary antibody (ThermoFisher Scientific) and Hoechst 33258 (10 μg/ml, Dojindo) in 2% (w/v) BSA in PBS for 1 h at RT. After three washes with PBS for 5 min each, the sections were mounted with SlowFade Gold antifade reagent (ThermoFisher Scientific). Images were obtained using an A1R+ confocal microscope (Nikon). For cultured cells, a primary antibody against H2B (1/400, Abcam) diluted in 2% (w/v) BSA in PBST was incubated with the cells for 1 h at RT. The cells were washed three times with PBST for 5 min each, and an Alexa Fluor 488-conjugated AffiniPure donkey anti-chicken IgY secondary antibody (IgG) (H + L) (1/200, Wako, Osaka, Japan) diluted in 2% (w/v) BSA in PBST was applied to the cells for 1 h at RT. The cells were subsequently washed three times for 5 min each with PBST. Next, a primary antibody against Hsp60 (1/400, Abcam) diluted in 2% (w/v) BSA in PBST was applied for 1 h at RT. The cells were washed three times with PBST for 5 min each, and highly cross-adsorbed Alexa Fluor 680-conjugated donkey anti-rabbit IgG (H + L) (1/200, ThermoFisher Scientific) diluted in 2% (w/v) BSA in PBST was applied to the cells for 1 h at RT. After three washes with PBST for 5 min each, images were obtained using an A1R+ confocal microscope (Nikon).

The probes (1 ng/μl) were also used for the electroporation of labeled mitochondrial DNA. The introduction of the probes to SH-SY5Y cells was performed using a NEPA21 electroporator (Nepagene; Poring pulse: 175 V; 2.5 ms; 50 ms interval; 2 pulses; 10% decay rate; + polarity, transfer pulse: 20 V; 50 ms; 50 ms interval; 5 pulses; 40% decay rate; +/− polarity).

### Laser microdissection

Paraffin sections (3 μm thick) were prepared before microdissection and mounted on PEN-membrane glass slides (Leica Microsystems). The sections were dewaxed and washed three times with PBS for 5 min each. The sections were incubated with 2% (w/v) BSA in PBS for 30 min. The primary antibodies against α-synuclein (1/500, ab155038, Abcam), used for DAB staining, and against α-synuclein (1/100, LB509, Abcam), used for immunofluorescence, were diluted in 2% (w/v) BSA in PBS and applied to the sections for 1 h at RT. The sections were washed three times with PBS for 5 min each. The following secondary antibodies were diluted in 2% (w/v) BSA in PBS and applied to the sections for 1 h at RT: biotinylated goat anti-rabbit IgG (1/500, Vector Laboratories) and DyLight 594-conjugated goat anti-mouse IgG (H + L) (1/100, ThermoFisher Scientific). The sections were washed three times with PBS for 5 min each. The sections used for DAB staining were further incubated with the VECTASTAIN Elite ABC HRP Kit reagent (Vector Laboratories) for 30 min, washed three times with PBS for 5 min each, developed using the ImmPACT DAB Peroxidase Substrate (Vector Laboratories), and washed three times with PBS for 5 min each. The sections were air-dried at RT immediately before laser microdissection, and 5 Lewy body cores were microdissected and collected in one tube. Regions of interest (ROIs) with the same shape were applied to the other controls. DNA was isolated using the QIAamp DNA FFPE Tissue Kit (Qiagen, Hilden, Germany). PCR was performed using Premix Taq (Ex Taq Version 2.0) (Takara Bio). qPCR was performed using TB Green Premix Ex Taq II (Takara Bio) and analyzed in a Thermal Cycler Dice Real Time System Lite (Takara Bio). The PCR primers used in the present study are listed in Supplementary Table 3.

### RNA sequencing

RNA sequencing was performed by Macrogen Japan (Kyoto, Japan). Libraries were prepared using the TruSeq RNA Sample Prep Kit v2 (Illumina, San Diego, CA, USA) according to the manufacturer’s guidelines. The libraries were sequenced on a NovaSeq 6000 instrument with 100-bp paired-end reads (Illumina).

### Statistics and reproducibility

Statistical analyses were performed using JMP software version 12.0 and 15.0 (JMP, Tokyo, Japan). Two-sided Student’s *t*-tests were used to compare arithmetic means between two groups. Pearson’s chi-squared test was applied to sets of categorical data. One-way ANOVA and post hoc analysis using Bonferroni’s correction for multiple tests were used unless indicated otherwise (Dunnett’s test was applied in some cases). Log-rank test was applied in survival analysis. Data are presented as the means ± standard errors of the means. *p* values < 0.05 were considered statistically significant. All the experiments associated with the main findings were replicated at least twice independently.

### Reporting summary

Further information on research design is available in the Nature Research Reporting Summary linked to this article.

## Supplementary information

Supplementary information

Reporting Summary

Description of Additional Supplementary Files

Supplementary Movie 1

Supplementary Movie 2

## Data Availability

All data that support the findings of this study are included in the manuscript or are available from the authors upon reasonable request. Source data are provided with this paper.

## Source data

Source Data
