# Supplementary material for: Cytosolic dsDNA of mitochondrial origin induces cytotoxicity and neurodegeneration in cellular and zebrafish models of Parkinson’s disease
Source: Nat Commun. 2021 May 25;12:3101. doi: 10.1038/s41467-021-23452-x (PMC8149644; doi:10.1038/s41467-021-23452-x)
Supplement: Supplementary file 1 — Supplementary information [file 41467_2021_23452_MOESM1_ESM.pdf]

Supplementary Information for

Cytosolic dsDNA of mitochondrial origin induces cytotoxicity and neurodegeneration in cellular and zebrafish models of Parkinson's disease

Hideaki Matsui,[1,2,#,*] Junko Ito,[3] Noriko Matsui,[1] Tamayo Uechi,[4] Osamu Onodera,[5] and Akiyoshi Kakita[3]

Hideaki Matsui

Email: hide0729@bri.niigata-u.ac.jp

This PDF file includes:

Supplementary Figures 1 to 5

Supplementary Tables 1 to 3

Other supplementary materials for this manuscript include the following:

Supplementary Movies 1 and 2

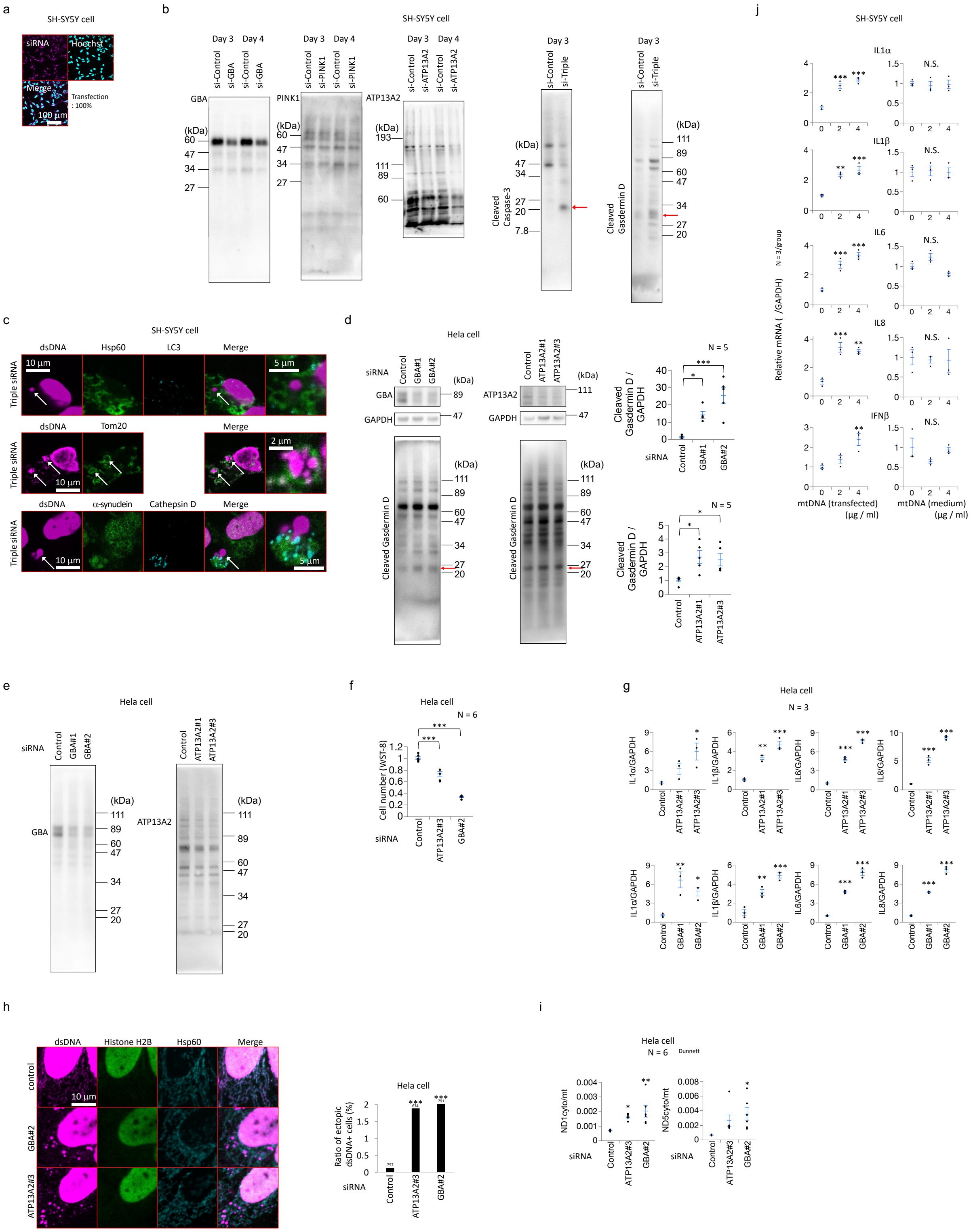
a
SH-SY5Y cell
siRNA
Hoechst
Merge
100 μm
Transfection : 100%
b
SH-SY5Y cell
Day 3
Day 4
si-Control
si-GBA
si-PINK1
si-ATP13A2
si-Triple
GBA
PINK1
ATP13A2
(kDa)
Cleaved Caspase-3
Cleaved Gasdermin D
c
SH-SY5Y cell
dsDNA
Hsp60
LC3
Merge
Tom20
α-synuclein
Cathepsin D
Triple siRNA
10 μm
5 μm
2 μm
d
Hela cell
siRNA
Control
GBA#1
GBA#2
ATP13A2#1
ATP13A2#3
GBA
GAPDH
ATP13A2
Cleaved Gasdermin D
N = 5
Cleaved Gasdermin D / GAPDH
e
Hela cell
f
Hela cell
N = 6
Cell number (WST-8)
g
Hela cell
N = 3
IL1α/GAPDH
IL1β/GAPDH
IL6/GAPDH
IL8/GAPDH
h
dsDNA
Histone H2B
Hsp60
Merge
control
GBA#2
ATP13A2#3
Hela cell
Ratio of ectopic dsDNA+ cells (%)
i
Hela cell
N = 6
Dunnett
ND1cyto/mt
ND5cyto/mt
j
SH-SY5Y cell
IL1α
IL1β
IL6
IL8
IFNβ
N.S.
N = 3/group
Relative mRNA (/GAPDH)
mtDNA (transfected) (μg / ml)
mtDNA (medium) (μg / ml)

Supplementary Fig. 1. Additional experiments related to Fig. 1 (a, b) Accell siRNA transfection efficiency in SH-SY5Y cells and full images of the Western blots shown in Fig. 1. (a) Accell siRNA transfection efficiency. An Accell Red nontargeting siRNA (Dharmacon) was used for the evaluation. (b) Full Western blots for GBA, ATP13A2 or PINK1 in the lysates of SH-SY5Y cells transfected with siRNAs. Full Western blot for cleaved caspase-3 and cleaved gasdermin D in the lysates of SH-SY5Y cells transfected with GBA, ATP13A2 and PINK1 siRNAs. si-Triple: Knockdown of GBA, ATP13A2 and PINK1 expression with siRNAs. (c) Additional images of immunofluorescence staining for cytosolic dsDNA in SH-SY5Y cells transfected with GBA, ATP13A2 and PINK1 siRNAs. Images of immunofluorescence staining for LC3, Hsp60, Tom20, cathepsin D or α-synuclein with cytosolic dsDNA are shown. White arrows indicate cytosolic dsDNA. Triple siRNA: Knockdown of GBA, ATP13A2 and PINK1 expression with siRNAs. (d-f) The death of GBA- or ATP13A2-depleted HeLa cells was determined using Western blotting for cleaved gasdermin D (N = 4 biologically independent samples. Data are presented as mean values +/- SEM. One-way ANOVA was followed by a post hoc analysis using two-sided Student's t-tests and Bonferroni's correction. GBA: ANOVA $p = 0.0003$, *: $p = 0.0079$, ***: $p < 0.0001$. ATP: ANOVA $p = 0.0153$, $p = 0.0081$ (Control vs. #1), $p = 0.0155$ (Control vs. #3)). (d, e) and the WST-8 assay (N = 6 biologically independent samples. Data are presented as mean values +/- SEM. One-way ANOVA was followed by a post hoc analysis using two-sided Student's t-tests and Bonferroni's correction. ANOVA $p < 0.0001$, ***: $p < 0.0001$). (f). (g) qPCR analyses of *IL-1α, IL-1β, IL-6* and *IL-8* mRNAs in GBA- or ATP13A2-depleted HeLa cells. N = 3 biologically independent samples. Data are presented as mean values +/- SEM. One-way ANOVA was followed by a post hoc analysis using Dunnett's test. ATP13A2 *IL-1α*: ANOVA $p = 0.0240$, *: $p = 0.0151$. *IL-1β*: ANOVA $p = 0.0001$, **: $p = 0.0011$, ***: $p < 0.0001$.

IL-6: ANOVA $p < 0.0001$, ***: $p < 0.0001$. *IL-8*: ANOVA $p < 0.0001$, ***: $p < 0.0001$. GBA *IL-1α*: ANOVA p = 0.0075, *: p = 0.0314, **: p = 0.0050. *IL-1β*: ANOVA p = 0.0003, **: p = 0.0047, ***: p = 0.0002. *IL-6*: ANOVA $p < 0.0001$, ***: p = 0.0001 (#1), ***: $p < 0.0001$ (#2). *IL-8*: ANOVA $p < 0.0001$, ***: $p < 0.0001$. (h) Immunostaining for dsDNA, histone H2B and Hsp60 in HeLa cells transfected with GBA or ATP13A2 siRNAs. The bar graph shows the ratio of ectopic dsDNA+ cells in HeLa cells transfected with GBA or ATP13A2 siRNAs. Pearson's chi-squared test was applied to sets of categorical data. The number on the graph indicates the total cell number counted (N = 757 cells for Control siRNA, N = 634 cells for ATP13A2 siRNA and N = 791 cells for GBA siRNA). (i) qPCR analysis of the *ND1* (Mitochondrially Encoded NADH:Ubiquinone Oxidoreductase Core Subunit 1) gene and *ND5* (Mitochondrially Encoded NADH:Ubiquinone Oxidoreductase Core Subunit 5) gene in the cytosolic fraction of GBA- or ATP13A2-depleted HeLa cells. N = 6 biologically independent samples. Data are presented as mean values +/- SEM. One-way ANOVA was followed by a post hoc analysis using Dunnett's test. ND1: ANOVA p = 0.0042, *: p = 0.0341, **: p = 0.0025. ND5: ANOVA p = 0.0277, *: p = 0.0177. cyto: Cytosolic fraction. mt: Mitochondrial fraction. (j) qPCR analysis of *IL-1α, IL-1β, IL-6, IL-8* and *IFN-β* mRNAs in SH-SY5Y cells in which mitochondrial DNA was introduced via electroporation. RNA was collected 1 day after electroporation. N = 3 biologically independent samples. Data are presented as mean values +/- SEM. One-way ANOVA was followed by a post hoc analysis using Dunnett's test. *IL-1α*: ANOVA p = 0.0002, p = 0.0005 (0 vs. 2), p = 0.0001 (0 vs. 4). *IL-1β*: ANOVA p = 0.0005, **: p = 0.0013, ***: p = 0.0005. *IL-6*: ANOVA p = 0.0003, p = 0.001 (0 vs. 2), p = 0.0002 (0 vs. 4). *IL-8*: ANOVA p = 0.0006, ***: p = 0.0006, **: p = 0.0011. *IFN-β*: ANOVA p = 0.009, **: p = 0.0064. N.S.: statistically not significant. Source data are provided as a Source Data file.

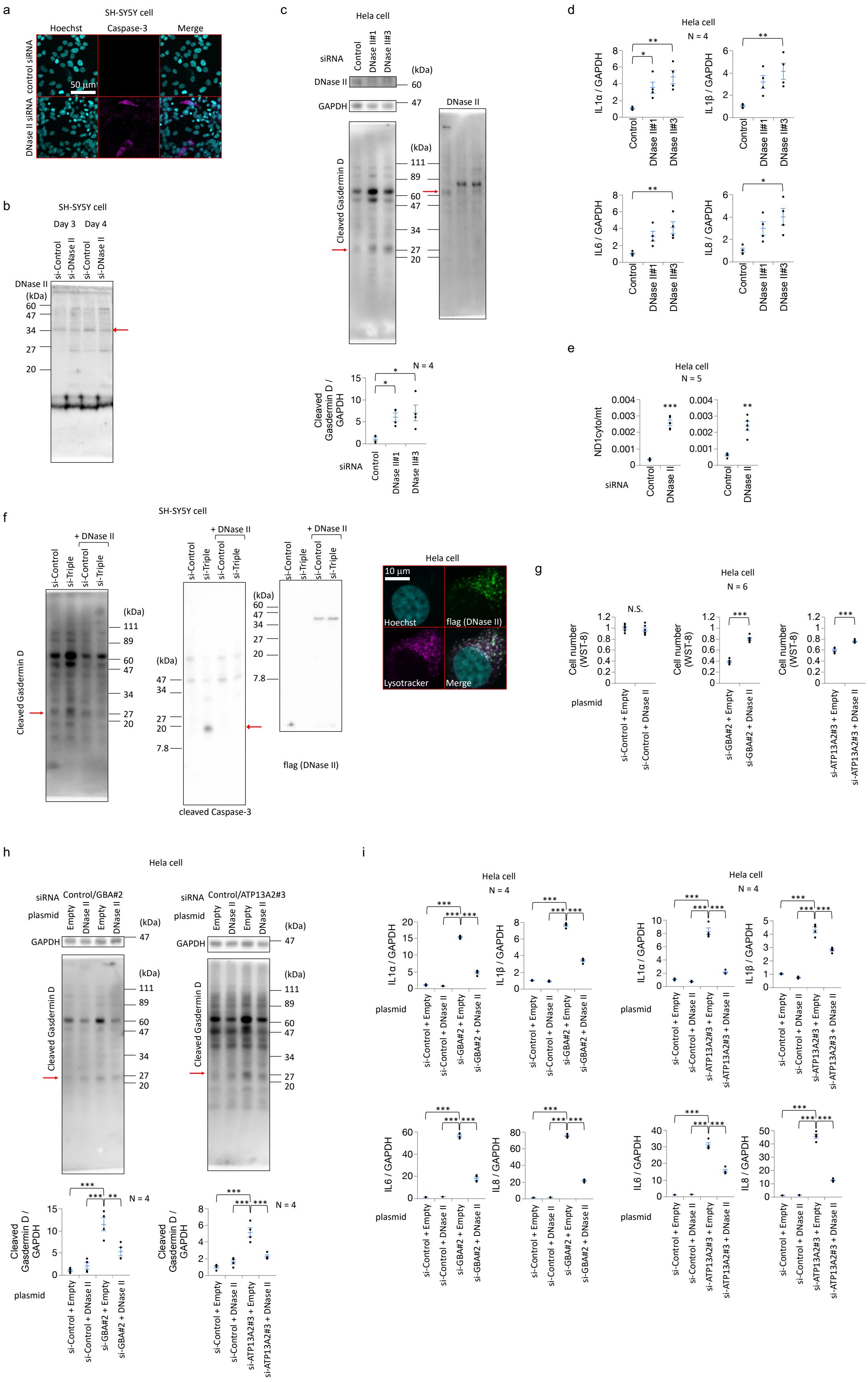
a
SH-SY5Y cell
Hoechst
Caspase-3
Merge
control siRNA
DNase II siRNA
50 μm
b
SH-SY5Y cell
Day 3
Day 4
si-Control
si-DNase II
DNase II
(kDa)
c
Hela cell
siRNA
Control
DNase II#1
DNase II#3
DNase II
GAPDH
(kDa)
Cleaved Gasdermin D
Cleaved Gasdermin D / GAPDH
N = 4
d
Hela cell
N = 4
IL1α / GAPDH
IL1β / GAPDH
IL6 / GAPDH
IL8 / GAPDH
e
Hela cell
N = 5
ND1cyto/mt
f
SH-SY5Y cell
+ DNase II
si-Control
si-Triple
Cleaved Gasdermin D
cleaved Caspase-3
flag (DNase II)
Hela cell
10 μm
Hoechst
flag (DNase II)
Lysotracker
Merge
g
Hela cell
N = 6
N.S.
Cell number (WST-8)
plasmid
si-Control + Empty
si-Control + DNase II
si-GBA#2 + Empty
si-GBA#2 + DNase II
si-ATP13A2#3 + Empty
si-ATP13A2#3 + DNase II
h
Hela cell
siRNA
Control/GBA#2
Control/ATP13A2#3
plasmid
Empty
DNase II
GAPDH
Cleaved Gasdermin D
Cleaved Gasdermin D / GAPDH
N = 4
i
Hela cell
N = 4
IL1α / GAPDH
IL1β / GAPDH
IL6 / GAPDH
IL8 / GAPDH
plasmid

Supplementary Fig. 2. Additional experiments related to Fig. 2. (a, b) Effect of DNase II knockdown on cell death. (a) Caspase-3 immunofluorescence in SH-SY5Y cells transfected with DNase II siRNA. (b) Full Western blot shown in Fig. 2a. (c) Western blot showing cleaved gasdermin D in HeLa cells transfected with DNase II siRNA. N = 4 biologically independent samples. Data are presented as mean values +/- SEM. One-way ANOVA was followed by a post hoc analysis using two-sided Student's t-tests and Bonferroni's correction. ANOVA $p = 0.0142$, $p = 0.0166$ (Control vs. #1), $p = 0.0067$ (Control vs. #3). (d) qPCR analysis of *DNase II, IL-1α, IL-1β, IL-6* and *IL-8* mRNAs in DNase II-depleted HeLa cells. N = 4 biologically independent samples. Data are presented as mean values +/- SEM. One-way ANOVA was followed by a post hoc analysis using two-sided Student's t-tests and Bonferroni's correction. *IL-1α*: ANOVA $p = 0.0048$, *: $p = 0.0163$, **: $p = 0.0016$. *IL-1β*: ANOVA $p = 0.0068$, **: $p = 0.0024$. *IL-6*: ANOVA $p = 0.0085$, **: $p = 0.0029$. *IL-8*: ANOVA $p = 0.0122$, *: $p = 0.0042$. (e) qPCR analysis of the *ND1* (Mitochondrially Encoded NADH:Ubiquinone Oxidoreductase Core Subunit 1) gene and *ND5* (Mitochondrially Encoded NADH:Ubiquinone Oxidoreductase Core Subunit 5) gene in the cytosolic fraction of DNase II-depleted HeLa cells. N = 5 biologically independent samples. Data are presented as mean values +/- SEM. Two-sided Student's t-tests were used. ***: $p = 0.0001$, **: $p = 0.0019$. cyto: Cytosolic fraction. mt: Mitochondrial fraction. (f) Full Western blots for cleaved caspase-3, cleaved gasdermin D and flag (DNase II) shown in Fig. 2d. Overexpressed DNase II mainly localized in lysosomes. si-Triple: Knockdown of GBA, ATP13A2 and PINK1 expression with siRNAs. (g-i) Effect of DNase II overexpression in HeLa cells transfected with the GBA or ATP13A2 siRNA. The WST-8 assay (N = 6 biologically independent samples. Data are presented as mean values +/- SEM. Two-sided Student's t-tests were used. si-GBA#2: $p < 0.0001$, si-ATP13A2#3: $p = 0.0002$) (g), a Western blot showing

cleaved gasdermin D (N = 4 biologically independent samples. Data are presented as mean values +/- SEM. One-way ANOVA was followed by a post hoc analysis using two-sided Student's t-tests and Bonferroni's correction. si-GBA#2: ANOVA $p < 0.0001$, ** : $p = 0.0007$, ***: $p < 0.0001$. si-ATP13A2#3: ANOVA $p < 0.0001$, ***: $p < 0.0001$) (h) and the qPCR results for *IL-1α, IL-1β, IL-6* and *IL-8* (N = 4 biologically independent samples. Data are presented as mean values +/- SEM. One-way ANOVA was followed by a post hoc analysis using two-sided Student's t-tests and Bonferroni's correction. si-GBA#2 *IL-1α*: ANOVA $p < 0.0001$, ***: $p < 0.0001$. si-GBA#2 *IL-1β*: ANOVA $p < 0.0001$, ***: $p < 0.0001$. si-GBA#2 *IL-6*: ANOVA $p < 0.0001$, ***: $p < 0.0001$. si-GBA#2 *IL-8*: ANOVA $p < 0.0001$, ***: $p < 0.0001$. si-ATP13A2#3 *IL-1α*: ANOVA $p < 0.0001$, ***: $p < 0.0001$. si- ATP13A2#3 *IL-1β*: ANOVA $p < 0.0001$, ***: $p < 0.0001$. si- ATP13A2#3 *IL-6*: ANOVA $p < 0.0001$, ***: $p < 0.0001$. si- ATP13A2#3 *IL-8*: ANOVA $p < 0.0001$, ***: $p < 0.0001$) (i) are shown. N.S.: statistically not significant. Source data are provided as a Source Data file.

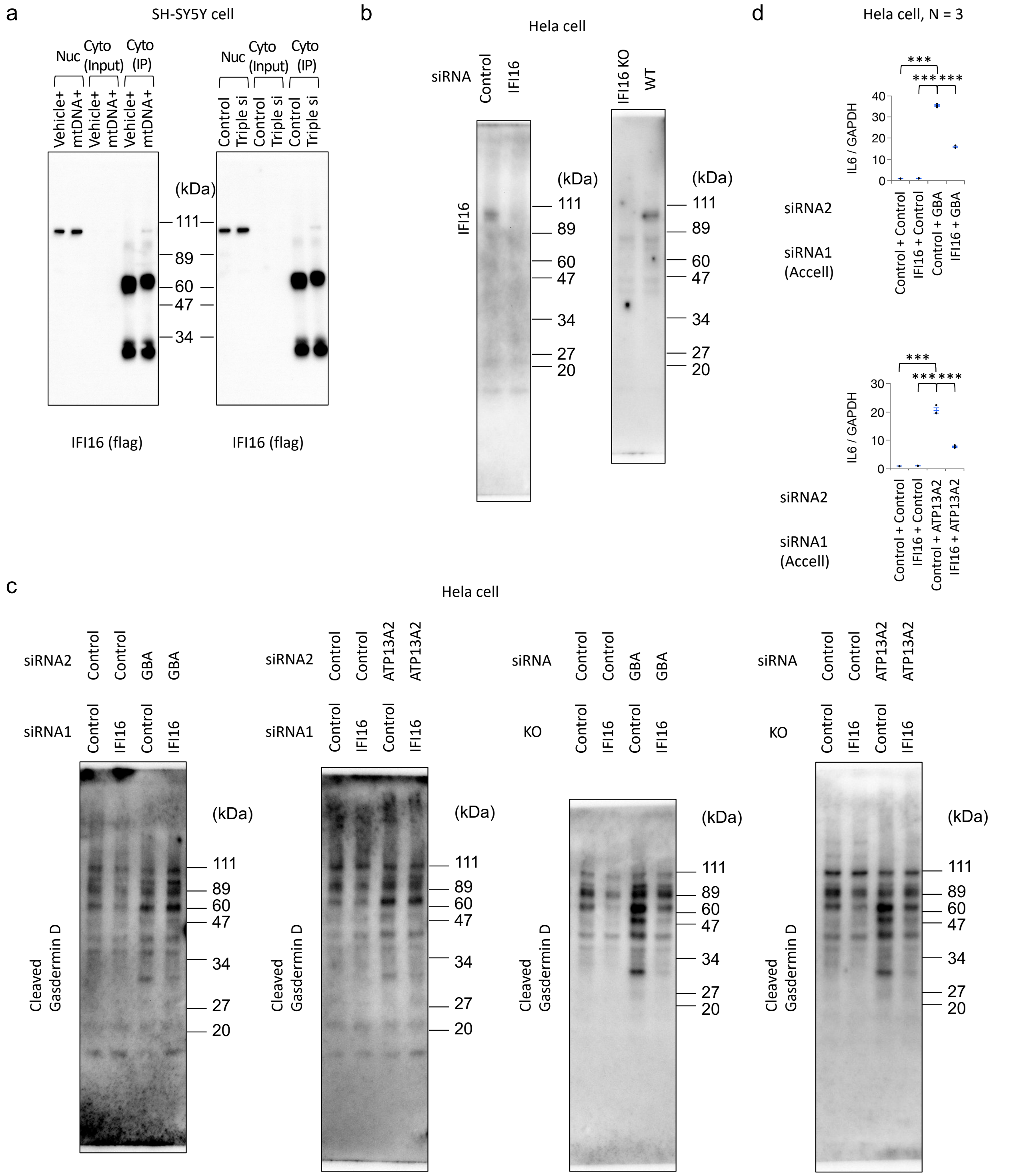

Supplementary Fig. 3. Additional experiments related to Fig. 3. (a) Full Western blot shown in Fig. 3a. Nuc: Nuclear fraction. Cyto: Cytosolic fraction. IP: Immunoprecipitation. (b) Full Western blot shown in Fig. 3d. WT: Wild type. KO: Knockout. (c) Full Western blot shown in

Fig. 3f. KO: Knockout. (d) Effect of IFI16 depletion (Accell siRNA knockdown) on type I IFN responses in HeLa cells with GBA or ATP13A2 knockdown. qPCR results for *IL-6* mRNAs are shown. N = 3 biologically independent samples. Data are presented as mean values +/- SEM. One-way ANOVA was followed by a post hoc analysis using two-sided Student's t-tests and Bonferroni's correction. GBA siRNA: ANOVA $p < 0.0001$, ***: $p < 0.0001$. ATP13A2 siRNA: ANOVA $p < 0.0001$, ***: $p < 0.0001$. Source data are provided as a Source Data file.

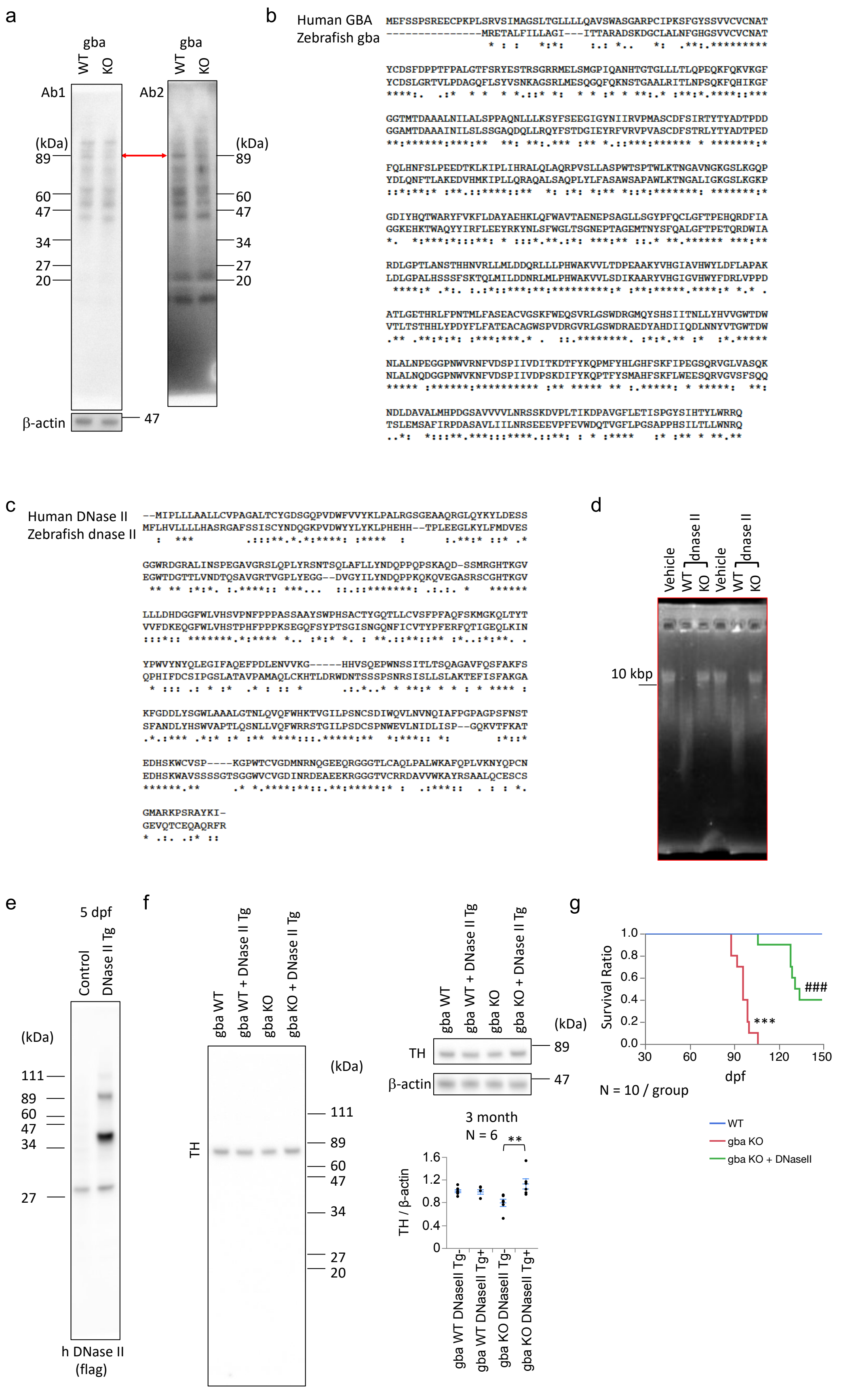
a
gba
WT
KO
Ab1
Ab2
(kDa)
89
60
47
34
27
20
β-actin
47
b
Human GBA
Zebrafish gba
c
Human DNase II
Zebrafish dnase II
d
Vehicle
WT
KO
dnase II
10 kbp
e
5 dpf
Control
DNase II Tg
(kDa)
111
89
60
47
34
27
h DNase II
(flag)
f
gba WT
gba WT + DNase II Tg
gba KO
gba KO + DNase II Tg
TH
(kDa)
111
89
60
47
34
27
20
β-actin
3 month
N = 6
**
TH / β-actin
1.6
1.2
0.8
0.4
0
gba WT DNaseII Tg-
gba WT DNaseII Tg+
gba KO DNaseII Tg-
gba KO DNaseII Tg+
g
Survival Ratio
1.0
0.8
0.6
0.4
0.2
0.0
30
60
90
120
150
dpf
***
###
N = 10 / group
WT
gba KO
gba KO + DNaseII

Supplementary Fig. 4. Additional experiments related to Fig. 4. (a) Full Western blot of the gba protein in gba WT and KO zebrafish brains. Ab1: anti-GBA antibody (ab55080, Abcam), Ab2: anti-GBA antibody (Novus Biologicals, NBP1-32271). WT: Wild type. KO: Knockout. (b) Comparison of the amino acid sequences of human GBA and zebrafish gba using ClustalOmega (http://www.clustal.org/omega). (c) Comparison of the amino acid sequences of human DNase II and zebrafish DNase II using ClustalOmega. (d) Assessment of the loss of DNase II enzyme activity related to mitochondrial DNA degradation in DNase II KO zebrafish at 12 months. WT: Wild type. KO: Knockout. (e) Full Western blot of DNase II (flag) shown in Fig. 4g. Tg: Transgenic. (f) Western blot of TH in gba KO zebrafish with or without the overexpression of human DNase II. N = 6 fish. Data are presented as mean values +/- SEM. Data are presented as mean values +/- SEM. One-way ANOVA was followed by a post hoc analysis using two-sided Student's t-tests and Bonferroni's correction. ANOVA $p = 0.0063$, **: $p = 0.0006$. WT: Wild type. KO: Knockout. Tg: Transgenic. TH: Tyrosine hydroxylase. (g) Survival curve of gba KO zebrafish with or without the overexpression of human DNase II. N = 10 fish. Log-rank test was applied in survival analysis. ***: $P < 0.0001$ (WT vs gba KO), ###: $P < 0.0001$ (gba KO vs gba KO + DNaseII). WT: Wild type. KO: Knockout. Source data are provided as a Source Data file.

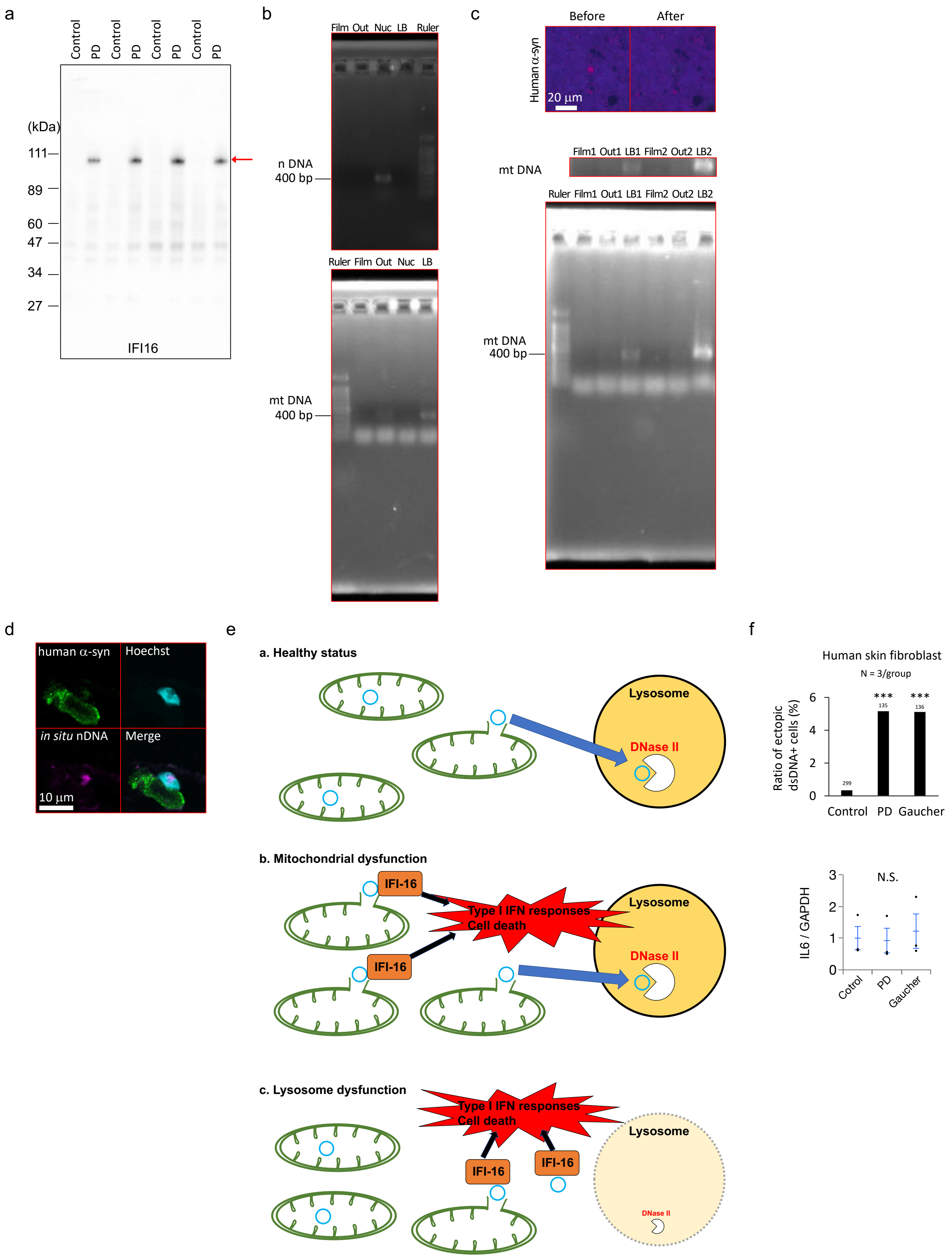
a
Control
PD
Control
PD
Control
PD
Control
PD
(kDa)
111
89
60
47
34
27
IFI16
b
Film Out Nuc LB Ruler
n DNA
400 bp
Ruler Film Out Nuc LB
mt DNA
400 bp
c
Before
After
Human α-syn
20 μm
mt DNA
Film1 Out1 LB1 Film2 Out2 LB2
Ruler Film1 Out1 LB1 Film2 Out2 LB2
mt DNA
400 bp
d
human α-syn
Hoechst
in situ nDNA
Merge
10 μm
e
a. Healthy status
Lysosome
DNase II
b. Mitochondrial dysfunction
IFI-16
Type I IFN responses
Cell death
Lysosome
IFI-16
DNase II
c. Lysosome dysfunction
Type I IFN responses
Cell death
Lysosome
IFI-16
IFI-16
DNase II
f
Human skin fibroblast
N = 3/group
Ratio of ectopic dsDNA+ cells (%)
***
***
Control
PD
Gaucher
IL6 / GAPDH
N.S.
Cotrol
PD
Gaucher

Supplementary Fig. 5. Additional experiments related to Fig. 5. (a) Full Western blot of IFI16 shown in Fig. 5c. PD: Parkinson's disease. (b) Full electrophoresis images of the PCR data shown in Fig. 5f. Film: samples dissected from only the cover film, Out: samples dissected from areas without Lewy bodies in the brain specimens, Nuc: samples dissected from the nuclei in the brain specimens (HE staining), LB: samples dissected from the cores of Lewy bodies in the brain specimens. mt DNA: Mitochondrial DNA. n NDA: Nuclear DNA. (c) Laser microdissection of brain sections and subsequent PCR amplification of mitochondrial sequences using immunofluorescence sections. "Before" shows immunofluorescence staining performed just before laser microdissection, and "After" shows staining after microdissection. Film: samples dissected from only the cover film, Out: samples dissected from Lewy bodies in the brain specimens, LB: samples dissected from the cores of Lewy bodies in the brain specimens. mt DNA: Mitochondrial DNA. α-syn: α-synuclein. (d) *In situ* hybridization of human brain tissues (medulla oblongata) using nuclear DNA probes. α-syn: α-synuclein. (e) Schematic illustration of the hypothesis. If the mitochondria are damaged (e-b) and/or autophagy-lysosome functions are impaired (e-c), the mitochondrial DNA, which should be rapidly degraded under normal conditions (e-a), persists in the cytosol and exerts toxic effects via the IFI16 protein. (f) The ratio of ectopic dsDNA+ cells and qPCR results for *IL-6* mRNAs (N = 3 biologically independent samples. Data are presented as mean values +/- SEM. One-way ANOVA was followed by a post hoc analysis using two-sided Student's t-tests and Bonferroni's correction) in human skin fibroblast from control, PD patients or Gaucher's disease patients. Pearson's chi-squared test was applied to sets of categorical data. The number on the graph indicates the total cell number counted (N = 299 cells for Control, N = 135 cells for PD and N = 136 cells for Gaucher). PD:

Parkinson's disease. Gaucher: Gaucher's disease. N.S.: statistically not significant. Source data are provided as a Source Data file.

Control	Catalog ID	Age	Gender
1	GM00041	0.25	Female
2	GM01680	71	Female
3	GM02912	26	Male
PD	Catalog ID	Age	Gender
1	AG08395	85	Female
2	AG20442	53	Male
3	AG20443	71	Male
Gaucher's disease	Catalog ID	Age	Gender
1	GM08760	1	Male
2	GM20270	0.33	Female
3	GM20273	No data	Male

Supplementary Table 1. Fibroblast profiles from Coriell Institute (Camden, NJ, USA).

PD	Age	Diease duration (year)	Experiments in this study
1	81	19	Imunohistochemistry, in situ hybridization
2	79	25	Imunohistochemistry, in situ hybridization, Biochemistry
3	83	17	Imunohistochemistry, in situ hybridization, Biochemistry
4	81	15	Imunohistochemistry, in situ hybridization
5	87	10	Imunohistochemistry, in situ hybridization, Biochemistry
6	72	15	Imunohistochemistry, in situ hybridization
7	87	10	Biochemistry
Control	Age	Diagnosis	Experiments in this study
1	80	Abdominal hemorrhage	Imunohistochemistry, in situ hybridization
2	76	Visceral vasculitis	Imunohistochemistry, in situ hybridization
3	79	Foix-Alajouanine syndrome	Imunohistochemistry, in situ hybridization
4	76	Lambert-Eaton	Imunohistochemistry, in situ hybridization
5	78	Myopathy	Imunohistochemistry, in situ hybridization
6	75	Cushing syndrome	Imunohistochemistry, in situ hybridization
7	66	Pellagra encephalopathy	Biochemistry
8	62	Myotonic dystrophy	Biochemistry
9	75	Pancreatic cancer, Brain metastasis	Biochemistry
10	83	Chronic inflammatory demyelinating polyneuropathy	Biochemistry

Supplementary Table 2. Patient profiles. Information on the patients included in this study.

Antibody	Company	ID
Anti-PINK1 antibody	Abcam	Cat# ab23707, RRID:AB_447627
Anti-GBA antibody	Novus Biologicals	Cat# NBP1-32271, RRID:AB_2109073
Anti-GBA antibody	Abcam	Cat# ab55080, RRID:AB_2109076
Anti-ATP13A2 Antibody	Novus Biologicals	Cat# NB110-41486SS, RRID:AB_1290620
Anti-DNase II antibody	Abcam	Cat# ab8119, RRID:AB_306289
Anti-α-synuclein antibody [MJFR1]	Abcam	Cat# ab138501, RRID:AB_2537217
Anti-α-synuclein antibody [LB 509]	Abcam	Cat# ab27766, RRID:AB_727020
Anti-active Caspase-3 antibody [E83-77]	Abcam	Cat# ab32042, RRID:AB_725947
Anti-Caspase-3 antibody	Abcam	Cat# ab13847, RRID:AB_443014
Anti-cleaved Gasdermin D antibody [E7H9G]	Cell Signaling Technology	Cat# 36425, RRID:AB_2799099
Monoclonal anti-β-Actin antibody produced in mouse clone AC-15	Sigma-Aldrich	Cat# A5441, RRID:AB_476744
Anti-GAPDH antibody	Wako	Cat# 016-25523, RRID:AB_2814991
Anti-Hsp60 antibody	Abcam	Cat# ab46798, RRID:AB_881444
Anti-Histone H2B antibody	Abcam	ab134211
Anti-LC3 mAb	MBL International	Cat# M152-3, RRID:AB_1279144
Anti-dsDNA antibody [35I9 DNA]	Abcam	Cat# ab27156, RRID:AB_470907
Tom20 (D8T4N) Rabbit mAb	Cell Signaling Technology	Cat# 42406, RRID:AB_2687663
Anti-α-Synuclein Antibody	Novus Biologicals	NBP2-25146
Monoclonal ANTI-FLAG M2 antibody produced in mouse, 1 mg/mL, clone M2	Sigma-Aldrich	Cat# F1804, RRID:AB_262044
Anti-Cathepsin D (Ab-1) Mouse mAb (BC011)	Merck	Cat# IM03, RRID:AB_2087109
Anti-Tyrosine Hydroxylase Antibody	Merck	Cat# AB152, RRID:AB_390204
Anti-VDAC1 / Porin antibody - Mitochondrial Loading Control	Abcam	Cat# ab15895, RRID:AB_2214787
Anti-Phosphorylated α-Synuclein, Monoclonal Antibody (pSyn#64)	Wako	Cat# 014-20281, RRID:AB_516843
Anti-α-synuclein antibody	Abcam	ab155038
Anti-COX IV antibody [20E8C12]	Abcam	Cat# ab14744, RRID:AB_301443
Anti-DDDDK tag antibody	Abcam	Cat# ab1170, RRID:AB_298495
Anti-IFI16 antibody [1G7]	Santa Cruz Biotechnology (Dallas, TX, USA)	Cat# sc-8023, RRID:AB_627775
Anti-IFI16 antibody [EPR11767(B)]	Abcam	Cat# ab169788, RRID:AB_298495
Alexa Fluor 488-AffiniPure Donkey Anti-Chicken IgY (IgG) (H+L)	Wako	563-78311
Goat anti-Rabbit IgG (H+L) Cross-Adsorbed Secondary Antibody, Alexa Fluor 350	Thermo Fischer Scientific	Cat# A-11046, RRID:AB_2534101
Goat anti-Rabbit IgG (H+L) Cross-Adsorbed Secondary Antibody, Alexa Fluor 488	Thermo Fischer Scientific	Cat# A-11008, RRID:AB_143165
Goat anti-Rabbit IgG (H+L) Highly Cross-Adsorbed Secondary Antibody, Alexa Fluor 594	Thermo Fischer Scientific	Cat# A-11037, RRID:AB_2534095
Goat anti-Rabbit IgG (H+L) Cross-Adsorbed Secondary Antibody, Alexa Fluor 594	Thermo Fischer Scientific	Cat# A-11012, RRID:AB_2534079
Goat anti-Rabbit IgG (H+L) Highly Cross-Adsorbed Secondary Antibody, Alexa Fluor 680	Thermo Fischer Scientific	Cat# A-21109, RRID:AB_2535758
Goat anti-Mouse IgG (H+L) Cross-Adsorbed Secondary Antibody, Alexa Fluor 350	Thermo Fischer Scientific	Cat# A-11045, RRID:AB_2534100
Goat anti-Mouse IgG (H+L) Cross-Adsorbed Secondary Antibody, Alexa Fluor 488	Thermo Fischer Scientific	Cat# A-11001, RRID:AB_2534069
Goat anti-Mouse IgG (H+L) Cross-Adsorbed Secondary Antibody, Alexa Fluor 594	Thermo Fischer Scientific	Cat# A-11005, RRID:AB_2534073
Goat anti-Mouse IgG (H+L) Highly Cross-Adsorbed Secondary Antibody, Alexa Fluor 680	Thermo Fischer Scientific	Cat# A-21058, RRID:AB_2535724
Goat anti-Mouse IgG (H+L) Secondary Antibody, DyLight 594	Thermo Fischer Scientific	Cat# 35510, RRID:AB_1185569
Donkey anti-Mouse IgG (H+L) Highly Cross-Adsorbed Secondary Antibody, Alexa Fluor 350	Thermo Fischer Scientific	Cat# A10035, RRID:AB_2534011
Donkey anti-Goat IgG (H+L) Cross-Adsorbed Secondary Antibody, Alexa Fluor 488	Thermo Fischer Scientific	Cat# A-11055, RRID:AB_2534102
Donkey anti-Mouse IgG (H+L) Highly Cross-Adsorbed Secondary Antibody, Alexa Fluor 594	Thermo Fischer Scientific	Cat# A-21203, RRID:AB_141633
Donkey anti-Rabbit IgG (H+L) Highly Cross-Adsorbed Secondary Antibody, Alexa Fluor 680	Thermo Fischer Scientific	Cat# A10043, RRID:AB_2534018
Donkey Anti-Goat IgG H&L (Alexa Fluor 594)	Abcam	ab150132
Biotinylated goat anti-rabbit IgG	Vector Laboratories	Cat# BA-1000, RRID:AB_2313606
**Oligonucleotides**	**Company**	**ID**
IL1α Forward Primer for qPCR: TGTAAGCTATGGCCCACTCCA	Integrated DNA Technologies	N/A
IL1α Reverse Primer for qPCR: AGAGACACAGATTGATCCATGCA	Integrated DNA Technologies	N/A
IL1β Forward Primer for qPCR: CTCTCTCCTTTCAGGGCCAA	Integrated DNA Technologies	N/A
IL1β Reverse Primer for qPCR: GAGAGGCCTGGCTCAACAAA	Integrated DNA Technologies	N/A
IL6 Forward Primer for qPCR: CACCGGGAACGAAAGAGAAG	Integrated DNA Technologies	N/A
IL6 Reverse Primer for qPCR: TCATAGCTGGGCTCCTGGAG	Integrated DNA Technologies	N/A
IL8 Forward Primer for qPCR: ACATGACTTCCAAGCTGGCC	Integrated DNA Technologies	N/A
IL8 Reverse Primer for qPCR: CAGAAATCAGGAAGGCTGCC	Integrated DNA Technologies	N/A
MMP3 Forward Primer for qPCR: GGATGCCAGGAAAGGTTCTG	Integrated DNA Technologies	N/A
MMP3 Reverse Primer for qPCR: CCAGGTGTGGAGTTCCTGATGT	Integrated DNA Technologies	N/A
GAPDH Forward Primer for qPCR: CAGCCTCAAGATCATCAGCA	Integrated DNA Technologies	N/A
GAPDH Reverse Primer for qPCR: TGTGGTCATGAGTCCTTCCA	Integrated DNA Technologies	N/A
STING Forward Primer for qPCR: ATATCTGCGGCTGATCCTGC	Integrated DNA Technologies	N/A
STING Reverse Primer for qPCR: TTGTAAGTTCGAATCCGGGC	Integrated DNA Technologies	N/A
cGAS Forward Primer for qPCR: GGCGGTTTTGGAGAAGTTGA	Integrated DNA Technologies	N/A
cGAS Reverse Primer for qPCR: GCCGCCGTGGAGATATCAT	Integrated DNA Technologies	N/A
IFI16 Forward Primer for qPCR: CTACCCCAGGAACAGCGTCA	Integrated DNA Technologies	N/A
IFI16 Reverse Primer for qPCR: GGTTGTGCTGGCCTCTGAAG	Integrated DNA Technologies	N/A
PINK1 Forward Primer for qPCR: TACCAGTGCACCAGGAGAAG	Integrated DNA Technologies	N/A
PINK1 Reverse Primer for qPCR: GCTTGGGACCTCTCTTGGAT	Integrated DNA Technologies	N/A
ATP13A2 Forward Primer for qPCR: GTTATCCAGGCTCTGCGAAGGA	Integrated DNA Technologies	N/A
ATP13A2 Reverse Primer for qPCR: GTGGACGATGATCAGATGCTCC	Integrated DNA Technologies	N/A
GBA Forward Primer for qPCR: TGCTGCTCTCAACATCCTTGCC	Integrated DNA Technologies	N/A
GBA Reverse Primer for qPCR: TAGGTGCGGATGGAGAAGTCAC	Integrated DNA Technologies	N/A
DNASE2 Forward Primer for qPCR: CCTGCTCTACAATGACCAACCG	Integrated DNA Technologies	N/A
DNASE2 Reverse Primer for qPCR: GTACACTGTGGACCAGCCAGAA	Integrated DNA Technologies	N/A
IFNβ Forward Primer for qPCR: TTGTTGAGAACCTCCTGGCT	Integrated DNA Technologies	N/A
IFNβ Reverse Primer for qPCR: TGACTATGGTCCAGGCACAG	Integrated DNA Technologies	N/A
TNFα Forward Primer for qPCR: TCAGATCATCTTCTCGAACCCC	Integrated DNA Technologies	N/A
TNFα Reverse Primer for qPCR: ATCTCTCAGCTCCACGCCAT	Integrated DNA Technologies	N/A
Mitochondria genome Forward Primer for PCR: TGAAACTTCGGCTCACTCCT	Integrated DNA Technologies	N/A
Mitochondria genome Reverse Primer for PCR: AATGTATGGGATGGCGGATA	Integrated DNA Technologies	N/A
Chromosome 1 genome Forward Primer for PCR: TGGGGGAGAACAGACAAATC	Integrated DNA Technologies	N/A
Chromosome 1 genome Reverse Primer for PCR: AGGAGGGCCAGTCAGGTATT	Integrated DNA Technologies	N/A
Human Mitochondrial DNA (mtDNA) Monitoring Primer Set	Takara Bio	Cat# 7246
zebrafish gba Forward Primer for PCR and sequence: CGGAATAATCACCACAGCAA	Integrated DNA Technologies	N/A
zebrafish gba Reverse Primer for PCR and sequence: AAGAGCACTCACCTGCACCT	Integrated DNA Technologies	N/A
zebrafish dnase II Forward Primer for PCR and sequence: GCGGATTTCCATCATGTTTC	Integrated DNA Technologies	N/A
zebrafish dnase II Reverse Primer for PCR and sequence: GGCTCACATTGTCCTTTTAGG	Integrated DNA Technologies	N/A
Accell Red Non-targeting siRNA, 5 nmol	Dharmacon	Cat# D-001960-01-05
Accell Human PINK1 (65018) siRNA - SMARTpool, 50 nmol	Dharmacon	Cat# E-004030-00-0050
Accell Human ATP13A2 (23400) siRNA - SMARTpool, 50 nmol	Dharmacon	Cat# E-008601-00-0050
Accell Human GBA (2629) siRNA - SMARTpool, 50 nmol	Dharmacon	Cat# E-006366-00-0050
Accell Human DNASE2(1777) siRNA - SMARTpool, 50 nmol	Dharmacon	Cat# E-009667-00-0050
Accell Non-targeting Pool, 50 nmol	Dharmacon	Cat# D-001910-10-50
GBA siRNA #1	Thermo Fischer Scientific	Cat# HSS142157
GBA siRNA #2	Thermo Fischer Scientific	Cat# HSS142158
ATP13A2 siRNA #1	Thermo Fischer Scientific	Cat# HSS118711
ATP13A2 siRNA #3	Thermo Fischer Scientific	Cat# HSS177397
DNase II siRNA #1	Thermo Fischer Scientific	Cat# HSS102841
DNase II siRNA #3	Thermo Fischer Scientific	Cat# HSS102843
IFI16 siRNA #3	Thermo Fischer Scientific	Cat# HSS105207

Supplementary Table 3. List of antibodies and oligonucleotides used in this study.

Figure	Details of statistics
1a, a-1	qPCR, N = 3 biologically independent samples. Data are presented as mean values +/- SEM. Two-sided Student's t-tests were used. Control vs. GBA siRNA: p = 0.0009, Control vs. PINK1 siRNA: p = 0.0139, Control vs. ATP13A2 siRNA: p = 0.0001.
1a, a-2	N = 10 biologically independent samples. Data are presented as mean values +/- SEM. One-way ANOVA was followed by a post hoc analysis using two-sided Student's t-tests and Bonferroni's correction. LDH assay: ANOVA p < 0.0001, ***: p < 0.0001. WST-8 assay: ANOVA p < 0.0001, ***: p < 0.0001.
1a, a-3	N = 4 biologically independent samples. Data are presented as mean values +/- SEM. Two-sided Student's t-tests were used. p = 0.0092.
1b	N = 6 biologically independent samples for IFN-β. N = 3 biologically independent samples for others. Data are presented as mean values +/- SEM. One-way ANOVA was followed by a post hoc analysis using Dunnett's test. IL-1α: ANOVA p < 0.0001, *: p = 0.0131, **: p = 0.0049, ***: p < 0.0001. IL-1β: ANOVA p = 0.0658, *: p = 0.0294. MMP-3: ANOVA p = 0.0008, *: p = 0.0120 (GBA siRNA), *: p = 0.0324 (ATP13A2 siRNA), ***: p = 0.0003. IL-6: ANOVA p = 0.0143, *: p = 0.0102 (PINK1 siRNA), *: p = 0.0429 (GBA siRNA). IL-8: ANOVA p = 0.0075, *: p = 0.0028. IFN-β: ANOVA p = 0.0004, *: p = 0.0418, ***: p < 0.0001.
1d, d-1	Pearson's chi-squared test was applied to sets of categorical data. ***: p = 0.0001. The number on the graph indicates the total cell number counted (N = 1095 cells for Control siRNA and N = 897 cells for Triple siRNA).
1d, d-2	N = 8 sets of values. r = 0.9421.
2a, a-1	qPCR, N = 3 biologically independent samples. Data are presented as mean values +/- SEM. Two-sided Student's t-tests were used. ***: p = 0.0006.
2a, a-2	Pearson's chi-squared test was applied to sets of categorical data. ***: p < 0.0001. The number on the graph indicates the total cell number counted (N = 576 cells for Control siRNA and N = 431 cells for DNase II siRNA).
2a, a-3	Pearson's chi-squared test was applied to sets of categorical data. ***: p < 0.0001. The number on the graph indicates the total cell number counted (N = 6388 cells for Control siRNA and N = 5881 cells for DNase II siRNA).
2c	N = 3 biologically independent samples. Data are presented as mean values +/- SEM. Two-sided Student's t-tests were used. IL-1α: **: p = 0.0055. IL-1β: ***: p = 0.0002. IL-6: *: p = 0.0106. IFN-β: ***: p = 0.0010.
2d, d-1	Pearson's chi-squared test was applied to sets of categorical data. ***: p < 0.0001. The number on the graph indicates the total cell number counted (N = 1410 cells for Control siRNA + empty, N = 1528 cells for Control siRNA + DNase II, N = 1373 cells for Triple siRNA + empty and N = 1155 cells for Triple siRNA + DNase II).
2d, d-2	N = 4 biologically independent samples. Data are presented as mean values +/- SEM. One-way ANOVA was followed by a post hoc analysis using two-sided Student's t-tests and Bonferroni's correction. ANOVA p = 0.0009, *: p = 0.0024, **: p = 0.0014, ***: p = 0.0001.
	Pearson's chi-squared test was applied to sets of categorical data (PI). ***: p < 0.0001. The number on the graph indicates the total cell number counted (N = 594 cells for Control siRNA + empty, N = 690 cells for Triple siRNA + empty and N = 554 cells for Triple siRNA + DNase II).
2e	N = 4 biologically independent samples. Data are presented as mean values +/- SEM. One-way ANOVA was followed by a post hoc analysis using two-sided Student's t-tests and Bonferroni's correction. IL-1α: ANOVA p < 0.0001, ***: p < 0.0001. IL-1β: ANOVA p < 0.0001, ***: p < 0.0001. IL-6: ANOVA p < 0.0001, ***: p < 0.0001. IL-8: ANOVA p < 0.0001, ***: p < 0.0001.
3e	N = 6 biologically independent samples. Data are presented as mean values +/- SEM. Two-sided Student's t-tests were used. ***: p < 0.0001.
3f	N = 4 biologically independent samples. Data are presented as mean values +/- SEM. One-way ANOVA was followed by a post hoc analysis using two-sided Student's t-tests and Bonferroni's correction. IFI16 siRNA/GBA siRNA: ANOVA p = 0.0031, *: p = 0.081, **: p = 0.0011. IFI16 siRNA/ATP13A2 siRNA: ANOVA p = 0.0091, p = 0.0036 (Control/ATP13A2 vs. IFI16/Control), p = 0.037 (Control/ATP13A2 vs. Control/Control), p = 0.072 (Control/ATP13A2 vs. IFI16/ATP13A2). IFI16 KO/GBA siRNA: ANOVA p = 0.0003, p < 0.0001 (Control/GBA vs. IFI16/Control), p = 0.0002 (Control/GBA vs. Control/Control), p = 0.0006 (Control/GBA vs. IFI16/GBA). IFI16 KO/ATP13A2 siRNA: ANOVA p = 0.0002, p < 0.0001 (Control/ATP13A2 vs. IFI16/Control), p = 0.0002 (Control/ATP13A2 vs. Control/Control), p = 0.0004 (Control/ATP13A2 vs. IFI16/ATP13A2).
3g	N = 3 biologically independent samples. Data are presented as mean values +/- SEM. One-way ANOVA was followed by a post hoc analysis using two-sided Student's t-tests and Bonferroni's correction. IFI16 siRNA/GBA siRNA IL-1α: ANOVA p < 0.0001, ***: p < 0.0001. IL-1β: ANOVA p < 0.0001, ***: p < 0.0001. IL-6: ANOVA p < 0.0001, ***: p < 0.0001. IL-8: ANOVA p < 0.0001, ***: p < 0.0001. IFI16 siRNA/ATP13A2 siRNA IL-1α: ANOVA p < 0.0001, ***: p < 0.0001. IL-1β: ANOVA p < 0.0001, ***: p < 0.0001. IL-6: ANOVA p < 0.0001, ***: p < 0.0001. IL-8: ANOVA p < 0.0001, ***: p < 0.0001. IFI16 KO/GBA siRNA: IL-1α: ANOVA p < 0.0001, ***: p < 0.0001. IL-1β: ANOVA p < 0.0001, ***: p < 0.0001. IL-6: ANOVA p < 0.0001, ***: p < 0.0001. IL-8: ANOVA p < 0.0001, ***: p < 0.0001. IFI16 KO/ATP13A2 siRNA: IL-1α: ANOVA p < 0.0001, ***: p = 0.0002 for WT/ATP13A2 siRNA vs. IFI16 KO/ATP13A2 siRNA, p < 0.0001 for others. IL-1β: ANOVA p < 0.0001, ***: p < 0.0001. IL-6: ANOVA p < 0.0001, ***: p = 0.0002 for WT/ATP13A2 siRNA vs. IFI16 KO/ATP13A2 siRNA, p < 0.0001 for others. IL-8: ANOVA p < 0.0001, ***: p < 0.0001.
3h	N = 3 biologically independent samples. Data are presented as mean values +/- SEM. One-way ANOVA was followed by a post hoc analysis using two-sided Student's t-tests and Bonferroni's correction. GBA siRNA: ANOVA p < 0.0001, ***: p < 0.0001. ATP13A2 siRNA: ANOVA p < 0.0001, ***: p < 0.0001.
4a	N = 5 fish for WT and N = 4 fish for KO. Data are presented as mean values +/- SEM. Two-sided Student's t-tests were used. *: p = 0.0398.
4b	N = 6 fish. Data are presented as mean values +/- SEM. Two-sided Student's t-tests were used. ***: p < 0.0001.
4c, c-1	N = 3 fish. Data are presented as mean values +/- SEM. Two-sided Student's t-tests were used. p = 0.0416, 0.0078, 0.0187, 0.0141, 0.0036, 0.0342, 0.05, 0.0225, 0.0310, 0.0468, 0.026 and 0.0288 (from left to right).
4c, c-2	N = 4 fish. Data are presented as mean values +/- SEM. Two-sided Student's t-tests were used. **: p = 0.0017.
4d	N = 4 fish. Data are presented as mean values +/- SEM. Two-sided Student's t-tests were used. ***: p < 0.0001.
4e	N = 5 fish. Data are presented as mean values +/- SEM. Two-sided Student's t-tests were used. DA: p = 0.0003. NE: p < 0.0001.
4f, f-2	N = 5 fish. Data are presented as mean values +/- SEM. Two-sided Student's t-tests were used. *: p = 0.0175.
4h	N = 4 fish. Data are presented as mean values +/- SEM. Two-sided Student's t-tests were used. *: p = 0.0353.
4i	N = 6 fish. Data are presented as mean values +/- SEM. Two-sided Student's t-tests were used. DA: p = 0.0002. NE: p = 0.0001.
4j	N = 10 fish. Data are presented as mean values +/- SEM. Two-sided Student's t-tests were used. **: p = 0.0062.
5a	N = 4 biologically independent samples. Data are presented as mean values +/- SEM. One-way ANOVA was followed by a post hoc analysis using two-sided Student's t-tests and Bonferroni's correction. IL-1α: ANOVA p = 0.0005, p = 0.0002 (Control/GBA vs. IFI16/Control), p = 0.0003 (Control/GBA vs. IFI16/GBA), p = 0.0005 (Control/GBA vs. Control/Control). IL-1β: ANOVA p < 0.0001, ***: p < 0.0001. IL-6: ANOVA p = 0.0004, p = 0.0001 (Control/GBA vs. IFI16/Control), p = 0.0002 (Control/GBA vs. IFI16/GBA), p = 0.0037 (Control/GBA vs. Control/Control). IL-8: ANOVA p = 0.0075, p = 0.0013 (Control/GBA vs. IFI16/Control), p = 0.0066 (Control/GBA vs. IFI16/GBA), p = 0.0146 (Control/GBA vs. Control/Control). IFI16: ANOVA p < 0.0001, p < 0.0001 (Control/GBA vs. IFI16/GBA), p < 0.0001 (Control/GBA vs. IFI16/Control), p = 0.0001 (Control/Control vs. IFI16/GBA), p = 0.0006 (Control/Control vs. IFI16/Control). GBA: ANOVA p = 0.0055, p = 0.0019 (Control/Control vs. IFI16/GBA), p = 0.0027 (Control/Control vs. Control/GBA).
5b	N = 6 individuals. Data are presented as mean values +/- SEM. Two-sided Student's t-tests were used. **: p = 0.0078.
5c, c-1	N = 4 individuals. Data are presented as mean values +/- SEM. Two-sided Student's t-tests were used. **: p = 0.0025.
5d	Typical Lewy bodies (n = 89) with a halo + core structure were analyzed in 6 brain tissues (medulla oblongata) from patients with Parkinson's disease.
5f	N = 4 biologically independent samples. Data are presented as mean values +/- SEM. Two-sided Student's t-tests were used. *: p = 0.031.

Supplementary Table 4. Additional details of statistics in this study.
