# Supplementary material for: Cytosolic dsDNA of mitochondrial origin induces cytotoxicity and neurodegeneration in cellular and zebrafish models of Parkinson’s disease
Source: Nat Commun. 2021 May 25;12:3101. doi: 10.1038/s41467-021-23452-x (PMC8149644; doi:10.1038/s41467-021-23452-x)
Supplement: Supplementary file 2 — Reporting Summary [file 41467_2021_23452_MOESM2_ESM.pdf]

natureresearch

Corresponding author(s): Hideaki Matsui

Last updated by author(s): Apr 16, 2021

Reporting Summary

Nature Research wishes to improve the reproducibility of the work that we publish. This form provides structure for consistency and transparency in reporting. For further information on Nature Research policies, see Authors & Referees and the Editorial Policy Checklist .

Statistics

For all statistical analyses, confirm that the following items are present in the figure legend, table legend, main text, or Methods section.

n/a	Confirmed	
☐	☒	The exact sample size (*n*) for each experimental group/condition, given as a discrete number and unit of measurement
☐	☒	A statement on whether measurements were taken from distinct samples or whether the same sample was measured repeatedly
☐	☒	The statistical test(s) used AND whether they are one- or two-sided<br>*Only common tests should be described solely by name; describe more complex techniques in the Methods section.*
☐	☒	A description of all covariates tested
☐	☒	A description of any assumptions or corrections, such as tests of normality and adjustment for multiple comparisons
☐	☒	A full description of the statistical parameters including central tendency (e.g. means) or other basic estimates (e.g. regression coefficient) AND variation (e.g. standard deviation) or associated estimates of uncertainty (e.g. confidence intervals)
☐	☒	For null hypothesis testing, the test statistic (e.g. *F*, *t*, *r*) with confidence intervals, effect sizes, degrees of freedom and *P* value noted<br>*Give P values as exact values whenever suitable.*
☒	☐	For Bayesian analysis, information on the choice of priors and Markov chain Monte Carlo settings
☒	☐	For hierarchical and complex designs, identification of the appropriate level for tests and full reporting of outcomes
☐	☒	Estimates of effect sizes (e.g. Cohen's *d*, Pearson's *r*), indicating how they were calculated

Our web collection on statistics for biologists contains articles on many of the points above.

Software and code

Policy information about availability of computer code

Data collection	Western blot images were aquired with Biotools MISVSII 1.0.<br>Real Time quantitative PCR data was collected on Takara Thermal Cycler Dice Real Time System Lite software 5.11.<br>RNA sequencing data was collected on Illumina NovaSeq6000 platform by Macrogen Japan Corp.<br>Microscopy images were acquired with Nikon Imaging Software NIS Elements.<br>DNA gel electrophoresis images were acquired with Astec Imager.
Data analysis	ImageJ (Fiji) was used for image analysis.<br>JMP 12 and JMP 15 software were used for statistical analysis.

For manuscripts utilizing custom algorithms or software that are central to the research but not yet described in published literature, software must be made available to editors/reviewers. We strongly encourage code deposition in a community repository (e.g. GitHub). See the Nature Research guidelines for submitting code & software for further information.

Data

Policy information about availability of data

All manuscripts must include a data availability statement. This statement should provide the following information, where applicable:

- Accession codes, unique identifiers, or web links for publicly available datasets
- A list of figures that have associated raw data
- A description of any restrictions on data availability

All data that support the findings of this study are included in the manuscript or are available from the authors upon reasonable request. Source data are provided with this paper.

Field-specific reporting

Please select the one below that is the best fit for your research. If you are not sure, read the appropriate sections before making your selection.

☒ Life sciences ☐ Behavioural & social sciences ☐ Ecological, evolutionary & environmental sciences

For a reference copy of the document with all sections, see nature.com/documents/nr-reporting-summary-flat.pdf

Life sciences study design

All studies must disclose on these points even when the disclosure is negative.

Sample size	No statistical methods were used to determine the sample size. The sample size follows common standards (n =3 biological replicates) and similar publications in the field. Size is reported in legends for main and Supplementary figures.
Data exclusions	No data was excluded from the analyses.
Replication	All the experiments associated to the main findings were replicated at least twice independently.
Randomization	All the samples analyzed in the study were clearly recognizable. I.E. transfected samples will express a protein that can be followed by WB. For this reason randomization was not required while findings are supported by independent biological replicates.
Blinding	Counting the number of neurons was conducted in a blinded manner. For other experiments, blinding was not required or not possible due to the experimental setup.

Reporting for specific materials, systems and methods

We require information from authors about some types of materials, experimental systems and methods used in many studies. Here, indicate whether each material, system or method listed is relevant to your study. If you are not sure if a list item applies to your research, read the appropriate section before selecting a response.

Materials & experimental systems

n/a	Involved in the study	
☐	☒	Antibodies
☐	☒	Eukaryotic cell lines
☒	☐	Palaeontology
☐	☒	Animals and other organisms
☐	☒	Human research participants
☒	☐	Clinical data

Methods

n/a	Involved in the study	
☒	☐	ChIP-seq
☒	☐	Flow cytometry
☒	☐	MRI-based neuroimaging

Antibodies

Antibodies used

Anti-PINK1 antibody Abcam Cat# ab23707, RRID:AB_447627
Anti-GBA antibody Novus Biologicals Cat# NBP1-32271, RRID:AB_2109073
Anti-GBA antibody Abcam Cat# ab55080, RRID:AB_2109076
Anti-ATP13A2 Antibody Novus Biologicals Cat# NB110-41486SS, RRID:AB_1290620
Anti-DNase II antibody Abcam Cat# ab8119, RRID:AB_306289
Anti-α-synuclein antibody [MJFR1] Abcam Cat# ab138501, RRID:AB_2537217
Anti-α-synuclein antibody [LB 509] Abcam Cat# ab27766, RRID:AB_727020
Anti-active Caspase-3 antibody [E83-77] Abcam Cat# ab32042, RRID:AB_725947
Anti-Caspase-3 antibody Abcam Cat# ab13847, RRID:AB_443014
Anti-cleaved Gasdermin D antibody [E7H9G] Cell Signaling Technology Cat# 36425, RRID:AB_2799099
Monoclonal anti-β-Actin antibody produced in mouse clone AC-15 Sigma-Aldrich Cat# A5441, RRID:AB_476744
Anti-GAPDH antibody Wako Cat# 016-25523, RRID:AB_2814991
Anti-Hsp60 antibody Abcam Cat# ab46798, RRID:AB_881444
Anti-Histone H2B antibody Abcam ab134211
Anti-LC3 mAb MBL International Cat# M152-3, RRID:AB_1279144
Anti-dsDNA antibody [35I9 DNA] Abcam Cat# ab27156, RRID:AB_470907
Tom20 (D8T4N) Rabbit mAb Cell Signaling Technology Cat# 42406, RRID:AB_2687663
Anti-α-Synuclein Antibody Novus Biologicals NBP2-25146
Monoclonal ANTI-FLAG M2 antibody produced in mouse, 1 mg/mL, clone M2 Sigma-Aldrich Cat# F1804, RRID:AB_262044
Anti-Cathepsin D (Ab-1) Mouse mAb (BC011) Merck Cat# IM03, RRID:AB_2087109

Anti-Tyrosine Hydroxylase Antibody Merck Cat# AB152, RRID:AB_390204
Anti-VDAC1 / Porin antibody - Mitochondrial Loading Control Abcam Cat# ab15895, RRID:AB_2214787
Anti-Phosphorylated α-Synuclein, Monoclonal Antibody (pSyn#64) Wako Cat# 014-20281, RRID:AB_516843
Anti-α-synuclein antibody Abcam ab155038
Anti-COX IV antibody [20E8C12] Abcam Cat# ab14744, RRID:AB_301443
Anti-DDDDK tag antibody Abcam Cat# ab1170, RRID:AB_298495
Anti-IFI16 antibody [1G7] Santa Cruz Biotechnology (Dallas, TX, USA) Cat# sc-8023, RRID:AB_627775
Anti-IFI16 antibody [EPR11767(B)] Abcam Cat# ab169788, RRID:AB_298495
Alexa Fluor 488-AffiniPure Donkey Anti-Chicken IgY (IgG) (H+L) Wako 563-78311
Goat anti-Rabbit IgG (H+L) Cross-Adsorbed Secondary Antibody, Alexa Fluor 350 Thermo Fischer Scientific Cat# A-11046, RRID:AB_2534101
Goat anti-Rabbit IgG (H+L) Cross-Adsorbed Secondary Antibody, Alexa Fluor 488 Thermo Fischer Scientific Cat# A-11008, RRID:AB_143165
Goat anti-Rabbit IgG (H+L) Highly Cross-Adsorbed Secondary Antibody, Alexa Fluor 594 Thermo Fischer Scientific Cat# A-11037, RRID:AB_2534095
Goat anti-Rabbit IgG (H+L) Cross-Adsorbed Secondary Antibody, Alexa Fluor 594 Thermo Fischer Scientific Cat# A-11012, RRID:AB_2534079
Goat anti-Rabbit IgG (H+L) Highly Cross-Adsorbed Secondary Antibody, Alexa Fluor 680 Thermo Fischer Scientific Cat# A-21109, RRID:AB_2535758
Goat anti-Mouse IgG (H+L) Cross-Adsorbed Secondary Antibody, Alexa Fluor 350 Thermo Fischer Scientific Cat# A-11045, RRID:AB_2534100
Goat anti-Mouse IgG (H+L) Cross-Adsorbed Secondary Antibody, Alexa Fluor 488 Thermo Fischer Scientific Cat# A-11001, RRID:AB_2534069
Goat anti-Mouse IgG (H+L) Cross-Adsorbed Secondary Antibody, Alexa Fluor 594 Thermo Fischer Scientific Cat# A-11005, RRID:AB_2534073
Goat anti-Mouse IgG (H+L) Highly Cross-Adsorbed Secondary Antibody, Alexa Fluor 680 Thermo Fischer Scientific Cat# A-21058, RRID:AB_2535724
Goat anti-Mouse IgG (H+L) Secondary Antibody, DyLight 594 Thermo Fischer Scientific Cat# 35510, RRID:AB_1185569
Donkey anti-Mouse IgG (H+L) Highly Cross-Adsorbed Secondary Antibody, Alexa Fluor 350 Thermo Fischer Scientific Cat# A10035, RRID:AB_2534011
Donkey anti-Goat IgG (H+L) Cross-Adsorbed Secondary Antibody, Alexa Fluor 488 Thermo Fischer Scientific Cat# A-11055, RRID:AB_2534102
Donkey anti-Rabbit IgG (H+L) Highly Cross-Adsorbed Secondary Antibody, Alexa Fluor 594 Thermo Fischer Scientific Cat# A-21207, RRID:AB_141637
Donkey anti-Rabbit IgG (H+L) Highly Cross-Adsorbed Secondary Antibody, Alexa Fluor 680 Thermo Fischer Scientific Cat# A10043, RRID:AB_2534018
Donkey Anti-Goat IgG H&L (Alexa Fluor 594) Abcam ab150132
Biotinylated goat anti-rabbit IgG Vector Laboratories Cat# BA-1000, RRID:AB_2313606

Validation

Anti-PINK1 antibody Abcam Cat# ab23707, RRID:AB_447627: In the company website, the antibody was validated for WB in human. In our hands observed bands were at the expected and reported sizes, decreasing in signal if siRNAs were transfected.
Anti-GBA antibody Novus Biologicals Cat# NBP1-32271, RRID:AB_2109073: In the company website, the antibody was validated for WB in human and zebrafish. In our hands observed bands were at the expected and reported sizes, decreasing in signal if siRNAs were transfected.
Anti-GBA antibody Abcam Cat# ab55080, RRID:AB_2109076: In the company website, the antibody was validated for WB in human. The signals were completely abrogated in KO cell samples. In our hands observed bands were at the expected and reported sizes, decreasing in signal if siRNAs were transfected.
Anti-ATP13A2 Antibody Novus Biologicals Cat# NB110-41486SS, RRID:AB_1290620: In the company website, the antibody was validated for WB in human. In our hands observed bands were at the expected and reported sizes, decreasing in signal if siRNAs were transfected.
Anti-DNase II antibody Abcam Cat# ab8119, RRID:AB_306289: In the company website, the antibody was validated for WB in human. In our hands observed bands were at the expected and reported sizes, decreasing in signal if siRNAs were transfected.
Anti-α-synuclein antibody [MJFR1] Abcam Cat# ab138501, RRID:AB_2537217: In the company website, the antibody was validated for WB, ICC and IHC in human. The signals were completely abrogated in KO cell samples.
Anti-α-synuclein antibody [LB 509] Abcam Cat# ab27766, RRID:AB_727020: In the company website, the antibody was validated for WB in human.
Anti-active Caspase-3 antibody [E83-77] Abcam Cat# ab32042, RRID:AB_725947: In the company website, the antibody was validated for WB, ICC and IF in human. The signals were completely abrogated in KO cell samples.
Anti-Caspase-3 antibody Abcam Cat# ab13847, RRID:AB_443014: In the company website, the antibody was validated for WB, ICC and IF in human. The signals were completely abrogated in KO cell samples.
Anti-cleaved Gasdermin D antibody [E7H9G] Cell Signaling Technology Cat# 36425, RRID:AB_2799099: In the company website, the antibody was validated for WB in human.
Monoclonal anti-β-Actin antibody produced in mouse clone AC-15 Sigma-Aldrich Cat# A5441, RRID:AB_476744: In the company website, the antibody was validated for WB in human and carp. In our hands observed bands were at the expected and reported sizes also in zebrafish.
Anti-GAPDH antibody Wako Cat# 016-25523, RRID:AB_2814991: In the company website, the antibody was validated for WB in human. In our hands observed bands were at the expected and reported sizes also in zebrafish.
Anti-Hsp60 antibody Abcam Cat# ab46798, RRID:AB_881444: In the company website, the antibody was validated for WB, IHC, ICC and IF in human.

Anti-Histone H2B antibody Abcam ab134211: In the company website, the antibody was validated for WB, IHC, ICC and IF in human.
Anti-LC3 mAb MBL International Cat# M152-3, RRID:AB_1279144: In the company website, the antibody was validated for WB, IHC and ICC in human.
Anti-dsDNA antibody [35I9 DNA] Abcam Cat# ab27156, RRID:AB_470907: In the company website, the antibody was validated for IHC, ICC and IF.
Tom20 (D8T4N) Rabbit mAb Cell Signaling Technology Cat# 42406, RRID:AB_2687663: In the company website, the antibody was validated for WB, IHC, ICC and IF in human.
Anti-α-Synuclein Antibody Novus Biologicals NBP2-25146: In the company website, the antibody was validated for WB, IHC, ICC and IF in human.
Monoclonal ANTI-FLAG M2 antibody produced in mouse, 1 mg/mL, clone M2 Sigma-Aldrich Cat# F1804, RRID:AB_262044: In the company website, the antibody was validated for WB, IHC, ICC and IF in all species.
Anti-Cathepsin D (Ab-1) Mouse mAb (BC011) Merck Cat# IM03, RRID:AB_2087109: In the company website, the antibody was validated for WB and IHC in human.
Anti-Tyrosine Hydroxylase Antibody Merck Cat# AB152, RRID:AB_390204: In the company website, the antibody was validated for WB, IHC, ICC and IF in human. In our hands observed bands were at the expected and reported sizes also in zebrafish. Observed signals were at the expected localization also in zebrafish.
Anti-VDAC1 / Porin antibody - Mitochondrial Loading Control Abcam Cat# ab15895, RRID:AB_2214787: In the company website, the antibody was validated for WB, ICC and IF in human. In our hands observed signals were at the expected localization also in zebrafish.
Anti-Phosphorylated α-Synuclein, Monoclonal Antibody (pSyn#64) Wako Cat# 014-20281, RRID:AB_516843: In the company website, the antibody was validated for WB and IHC in human.
Anti-α-synuclein antibody Abcam ab155038: In the company website, the antibody was validated for WB, IHC, ICC and IF in human.
Anti-COX IV antibody [20E8C12] Abcam Cat# ab14744, RRID:AB_301443: In the company website, the antibody was validated for WB in human.
Anti-DDDDK tag antibody Abcam Cat# ab1170, RRID:AB_298495: In the company website, the antibody was validated for WB in all species.
Anti-IFI16 antibody [1G7] Santa Cruz Biotechnology (Dallas, TX, USA) Cat# sc-8023, RRID:AB_627775: In the company website, the antibody was validated for WB, IHC, ICC and IF in human.
Anti-IFI16 antibody [EPR11767(B)] Abcam Cat# ab169788, RRID:AB_298495: In the company website, the antibody was validated for WB, IHC, ICC and IF in human.

Eukaryotic cell lines

Policy information about cell lines

Cell line source(s)

HeLa cells were derived from cervical cancer cells from a female (ATCC, CCL-2). HEK293 cells were derived from kidney of a fetus and HEK293T cells are expressing SV40 large T antigen (RIKEN cell bank, Tsukuba, Japan). SH-SY5Y cells were subcloned from SK-N-SH cells, which was isolated from a bone marrow biopsy from a female with neuroblastoma (EC94030304-F0, DS Pharma Biomedical, Suita, Japan).

Authentication

None of the cell lines have been authenticated.

Mycoplasma contamination

The cell lines were not tested for mycoplasma contamination.

Commonly misidentified lines
(See ICLAC register)

No commonly misidentified cell lines were used.

Animals and other organisms

Policy information about studies involving animals; ARRIVE guidelines recommended for reporting animal research

Laboratory animals

The zebrafish AB strain (https://zfin.org/ZDB-GENO-960809-7) was used in this study. Zebrafish mutants were generated using the TALEN method. The TALEN target sites in gba and DNase II were designed at the TAL Effector Nucleotide Targeter (TALE-NT) website (https://tale-nt.cac.cornell.edu/). TALEN plasmids were assembled with the Golden Gate TALEN and TAL Effector kit 2.0 according to the protocol published previously by Addgene (Cambridge, MA, USA) with slight modifications. Repeat variable di-residue (RVD) modules were cloned into pCS2TAL3DD and pCS2TAL3RR to generate the left TALEN and right TALEN, respectively. The TALEN sites were TGCTGCCAGATGCTGGTCAGTTCTTATCATATGTCAGCAATAAAGCTGGCAGCA for gba and TTTTCCTCCATCTCATGTTATAATGATCAAGGGAAACCTGTTGACTGGTTTGTA for DNase II (spacer sequence underlined). The capped mRNAs for the left and right TALENs were generated from NotI-digested pCS2TAL3DD and pCS2TAL3RR plasmids using the mMESSAGE mMACHINE SP6 kit (Thermo Fisher Scientific). Zebrafish embryos were microinjected at the one-cell stage. Injected founders (F0s) were grown to adulthood and outcrossed to wild-type AB partners, and genomic DNA was extracted from individual F1 embryos for PCR amplification and direct sequencing to identify the germline transmission of the mutations. The F1 generation and subsequent generations were genotyped using PCR (gba: forward primer: CGGAATAATCACCACAGCAA, reverse primer: AAGAGCACTCACCTGCACCT, DNase II: forward primer: GCGGATTTCCATCATGTTTC, reverse primer: GGCTCACATTGTCCTTTTAGG) and direct sequencing (gba: CGGAATAATCACCACAGCAA, DNase II: GCGGATTTCCATCATGTTTC). Heterozygous mutant fish were crossed to obtain homozygous mutant (gba KO or DNase II KO) and control fish. The oligos used for PCR and sequencing are listed in the Supplementary Table S3.
Plasmid DNA (Xenopus neural-specific beta tubulin (NBT) promoter: human DNase II-flag IRES GFP) was microinjected into the soma of one-cell stage wild-type embryos (= P0 generation, mosaic) at a concentration of 25 ng/ml to generate human DNase II

	transgenic fish. For the generation of stable transgenic fish, transgene cassettes flanked by recognition sites for the Tol2 transposase were used, and the DNA was supplemented with 25 ng/ml mRNA encoding the Tol2 transposase to increase the genomic integration of the transgene cassette. The injected P0 generation was raised to adulthood and crossed with wild-type fish to screen embryos of the F1 generation for the inherited expression of the fluorescent protein under a fluorescence stereomicroscope (MZ10F, Leica Microsystems, Wetzlar, Germany).<br>All the experimental analyses of zebrafish were conducted at 3 months except for DNase activity and electron microscopic analysis of dnase II KO fish (1 month), counting the number of ectopic dsDNA dots in dnase II KO (2 months), western blotting of flag-tag in human DNaseII Tg fish (5 dpf) and survival analysis (everyday). At these stage sexual maturation was not evident in dnase II KO and gba KO, thus we did not consider sex for these animals. For the other fish, equal numbers of male and female were used.
Wild animals	No wild animals were used in this study.
Field-collected samples	No field collected samples were used in this study.
Ethics oversight	All animal experiments were performed in compliance with the protocol reviewed by the Institutional Animal Care and Use Committee and approved by the President of Niigata University (Permit Number: #28 Niigata Univ. Res.367-1).

Note that full information on the approval of the study protocol must also be provided in the manuscript.

Human research participants

Policy information about studies involving human research participants

Population characteristics	PD:<br>1. Age: 81(year) Gender: Female PMI: 4(hour) Disease duration: 19(year)<br>2. Age: 79(year) Gender: Male PMI: 5.5(hour) Disease duration: 25(year)<br>3. Age: 83(year) Gender: Male PMI: 3.5(hour) Disease duration: 17(year)<br>4. Age: 81(year) Gender: Female PMI: 3(hour) Disease duration: 15(year)<br>5. Age: 87(year) Gender: Female PMI: 5(hour) Disease duration: 10(year)<br>6. Age: 72(year) Gender: Female PMI: 3(hour) Disease duration: 15(year)<br>7. Age: 87(year) Gender: Female PMI: 3(hour) Disease duration: 10(year)<br>Control<br>1. Age: 80(year) Gender: Male PMI: 3(hour) Disease: Abdominal hemorrhage<br>2. Age: 76(year) Gender: Male PMI: 2(hour) Disease: Visceral vasculitis<br>3. Age: 79(year) Gender: Female PMI: 2.5(hour) Disease: Foix-Alajouanine syndrome<br>4. Age: 76(year) Gender: Male PMI: 3(hour) Disease: Lambert-Eaton<br>5. Age: 78(year) Gender: Male PMI: 6(hour) Disease: Myopathy<br>6. Age: 75(year) Gender: Female PMI: 15(hour) Disease: Cushing syndrome<br>7. Age: 66(year) Gender: Male PMI: 3(hour) Disease: Pellagra encephalopathy<br>8. Age: 62(year) Gender: Female PMI: 6(hour) Disease: Myotonic dystrophy<br>9. Age: 75(year) Gender: Female PMI: 5(hour) Disease: Pancreatic cancer, Brain metastasis<br>10. Age: 83(year) Gender: Male PMI: 3(hour) Disease: Chronic inflammatory demyelinating polyneuropathy
Recruitment	Postmortem brains of BRI (Brain Research Institute of Niigata University) resource were used.
Ethics oversight	The study using postmortem brain samples from human subjects was approved by the Ethical Review Boards of Niigata University (#2515).

Note that full information on the approval of the study protocol must also be provided in the manuscript.
