# Supplementary material for: Cytosolic dsDNA of mitochondrial origin induces cytotoxicity and neurodegeneration in cellular and zebrafish models of Parkinson’s disease
Source: Nat Commun. 2021 May 25;12:3101. doi: 10.1038/s41467-021-23452-x (PMC8149644; doi:10.1038/s41467-021-23452-x)
Supplement: Supplementary file 3 — Description of Additional Supplementary Files [file 41467_2021_23452_MOESM3_ESM.pdf]

Description of Additional Supplementary Files

File Name: Supplementary Movie 1.

Description: Swimming movement of gba KO zebrafish (3 months old).

File Name: Supplementary Movie 2.

Description: Swimming movement of gba KO + human DNase II Tg zebrafish (3 months old).
